# Activation and Reactivity of the Deubiquitinylase OTU Cezanne-2 from MD Simulations and QM/MM Calculations

**DOI:** 10.1021/acs.jcim.4c01964

**Published:** 2025-01-09

**Authors:** Metehan Ilter, Andrés M. Escorcia, Eric Schulze-Niemand, Michael Naumann, Matthias Stein

**Affiliations:** † Molecular Simulations and Design Group, Max Planck Institute for Dynamics of Complex Technical Systems, Sandtorstrasse 1, 39106 Magdeburg, Germany; ‡ Institute for Experimental Internal Medicine, Medical Faculty, Otto von Guericke University, Leipziger Straße 44, 39120 Magdeburg, Germany

## Abstract

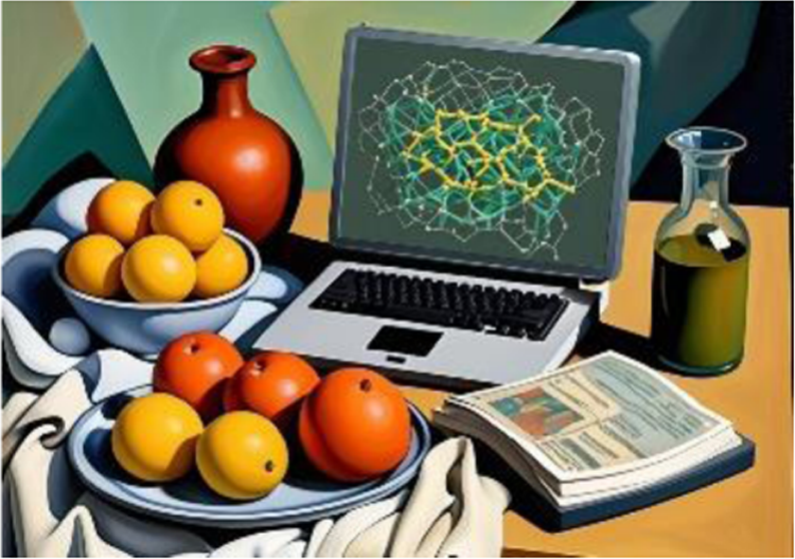

Cezanne-2 (Cez2) is a deubiquitinylating (DUB) enzyme involved in the regulation of ubiquitin-driven cellular signaling and selectively targets Lys11-linked polyubiquitin chains. As a representative member of the ovarian tumor (OTU) subfamily DUBs, it performs cysteine proteolytic isopeptide bond cleavage; however, its exact catalytic mechanism is not yet resolved. In this work, we used different computational approaches to get molecular insights into the Cezanne-2 catalytic mechanism. Extensive molecular dynamics (MD) simulations were performed for 12 μs to model free Cez2 and the diubiquitin (diUb) substrate-bound protein–protein complex in two different charge states of Cez2, each corresponding to a distinct reactive state in its catalytic cycle. The simulations were analyzed in terms of the relevant structural parameters for productive enzymatic catalysis. Reactive diUb–Cez2 complex configurations were identified, which lead to isopeptide bond cleavage and stabilization of the tetrahedral oxyanion intermediate. The reliability of these complexes was further assessed by quantum mechanics/molecular mechanics (QM/MM) optimizations. The results show that Cez2 follows a modified cysteine protease mechanism involving a catalytic Cys210/His367 dyad, with the oxyanion hole to be a part of the “C-loop,” and polarization of His367 by the formation of a strictly conserved water bridge with Glu173. The third residue has a dual role in catalysis as it mediates substrate binding and polarization of the catalytic dyad. A similar mechanism was identified for Cezanne-1, the paralogue of Cez2. In general, our simulations provide valuable molecular information that may help in the rational design of selective inhibitors of Cez2 and closely related enzymes.

## 1 Introduction

The covalent attachment of ubiquitin molecules (Ub) to target proteins, either as monoubiquitin (monoUb) or polyubiquitin (polyUb) chains, is a reversible post-translational modification in eukaryotes.^1^ This process is known as ubiquitinylation; PolyUb chains are formed through isopeptide bonds between the C-terminal glycine (G76) of a Ub chain (referred to as the “distal” Ub) and a lysine side chain (K6, K11, K27, K29, K33, K48, or K63) or the N-terminal methionine (M1) of a subsequent Ub (referred to as the “proximal” Ub). Different Ub-linkages play distinct roles in cellular signaling and disease biology, presenting new therapeutic opportunities.^2^

DUBs are proteases that counteract protein ubiquitinylation, thereby maintaining cellular ubiquitin homeostasis and controlling several cellular pathways, such as proteasome-mediated protein degradation, DNA damage response, and innate immune signaling.^3−8^ Dysregulation of DUBs is responsible for various diseases, and thus, DUBs are currently becoming recognized as attractive drug targets.^9−12^

The human genome encodes several DUB superfamilies, including 58 ubiquitin-specific proteases (USPs), 4 ubiquitin C-terminal hydrolyases (UCHs), 5 Machado-Josephin domain proteases (MJDs), 14 zinc metalloproteases (JAMMs), and 14 ovarian tumor domain proteases (OTUs). Of these, 79 DUBs are functional.^13^ OTU DUBs are cysteine proteases, which usually use a catalytic triad consisting of a cysteine/histidine pair and a third residue (such as aspartate or asparagine) to perform selective Ub-linkage recognition and cleavage. The role of the third residue, however, may vary.^14,15^ Deprotonation of the central cysteine residue by the nearby histidine, followed by a nucleophilic attack of the thiolate to the isopeptide ubiquitin linkage, leads to bond cleavage.^12,16^ Similar to the human genome, bacterial and viral genomes also encode OTUs, which can hijack human DUBs to circumvent the host’s immune response.^17−20^

OTU Cezanne-1 (OTUD7B; Cez) was isolated in 2001^21^ and first functionally characterized by Evans et al.^22^ Cezanne-1 is known to specifically perform the proteolysis of K11-linked polyubiquitin chains.^14^ Cezanne-1 plays a critical role in regulating various cellular processes, including nuclear factor ‘kappa-light-chain-enhancer’ of activated B-cells (NF-κB) signaling, T-cell activation, and the homeostasis of hypoxia-inducible factor 1 α (HIF-1α) and HIF-2α.^23−28^ Cez performs its catalytic function via a cysteine/histidine catalytic dyad, with a glutamate residue (Glu157) close to the dyad and is crucial for its pronounced K11 selectivity.^29^ Its dysfunction and its overexpression have been implicated in several cancer types.^14,29−31^ All-atom MD simulations of Cez in its ubiquitin-free, diubiquitin, and monoubiquitin-bound states provided insight into the conformational dynamics associated with its catalytic activation and cycle. However, the detailed reaction mechanism of Cezanne-1 was not addressed.^32^

Cezanne-2 (OTUD7A) also belongs to the OTU subfamily and is a paralogue to Cezanne-1.^6,14,31,33^ Cezanne-2 consists of a ubiquitin-associated binding domain (UBA), a catalytic (OTU) domain, and an A20-like zinc finger domain. For the UBA domain of Cezanne-2, an NMR structure (PDB ID: 2L2D) is available (see www.rscb.org), while the 3D structure of the catalytic OTU domain remains unresolved. Cezanne-1 and Cezanne-2 are the only DUBs with a striking specificity for K11-polyUb chains.^14^ Genomic alterations in Cezanne-2 cause several diseases, such as intellectual disability, epilepsy, dystonia, muscular hypotonia, seizures, and Ewing sarcoma.^33−39^

Biomolecular simulations have significantly enhanced our understanding of the protein structure, function, dynamics, and mechanisms. They bridge experimental gaps and provide atomistic details that can be used for the rational design of inhibitors.^40^ The data-driven AlphaFold2 (AF2) model has recently emerged as a powerful tool to predict 3D structures of proteins and protein–protein complexes at near experimental resolution.^41^ Molecular dynamics (MD) simulations offer insights into protein conformational dynamics that are inaccessible through X-ray crystallography, e.g., loop rearrangements, structure flexibility, and conformational transitions between inactive and active states.^42^ MD simulations have been used to study conformational dynamics and catalysis of both human and bacterial DUBs^20,43−48^ but much less so of OTUs.^20,32^ OTULIN (ovarian tumor deubiquitinylase with linear linkage specificity) and its bacterial analogue RavD exhibited a high degree of similarity in their distal S1 ubiquitin binding sites but showed differences regarding the proximal S1′ site composition.^20^ However, computational investigations on Cezanne-2 in its apo- and substrate-bound states have not been reported yet.

Results from complementary computational simulations are presented that elaborate on similarities and differences between Cezanne-2 and its paralogue Cezanne-1. Structural and conformational dynamics of apo Cez2 (Cez2apo) and diubiquitin-bound Cez2 (Cez2Ub_2_) were investigated using MD simulations for a total of 12 μs. The equilibria between different charge states of the active site were characterized, and we investigated the involvement of a catalytic dyad or triad in the isopeptide bond cleavage. From the MD trajectories, critical parameters for productive enzyme–substrate configurations suitable for proteolytic diUb cleavage were identified based on carefully defined structural criteria and verified by QM/MM calculations. It was possible to assign a catalytic dyad of histidine and cysteine residues to be functionally involved, while the third glutamate residue assisted in substrate recognition and positioning. A strictly conserved water molecule was identified that mediated the interaction between the active site histidine and glutamate residues. For comparison, an additional 4 μs of MD simulations for Cez were performed and revealed the strictly conserved water bridge. Thus, this water-mediated stabilization of the active site along the Cezanne-1 and Cezanne-2 reaction mechanisms should be considered when targeting these OTU DUBs.

## 2 Methods

### 2.1 Protein Structure Generation and Preparation

**Figure 1:**
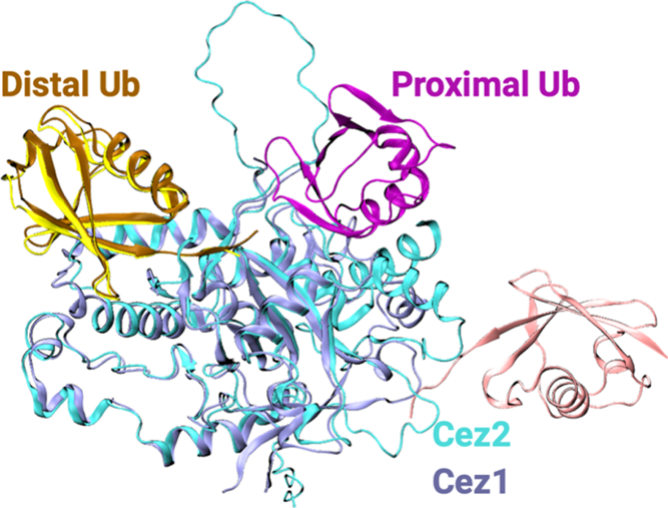
Structural comparison of models of diUb-bound Cezanne-2 with diUb in complex with Cezanne-1 (5LRV). OTU domains of Cezanne-1 and Cezanne-2 are colored ice blue and cyan, respectively. The distal Ub of Cezanne-2 is colored ocher, whereas that of Cezanne-1 is yellow. Proximal Ub atoms of Cezanne-1 and Cezanne-2 are depicted in purple and pink colors, respectively.

The amino acid sequence of human Cezanne-2 (UniProt ID: Q8TE49) was retrieved from the UniProt database, and AF2 (version 2.2.0) with default settings was used to generate a model of its 3D structure.^41,49^ The superimposition of the OTU domain of the top-ranked model with the crystal structure of the diUb-bound catalytic OTU domain of Cez (PDB ID: 5LRV) revealed a similar fold between these structures (see Figure 1).^29^ This agrees well with a sequence identity of 73.7% between the OTU domains of Cezanne-2 and Cezanne-1. In combination with a high per-residue model confidence score (pLDDT score of 88.59 ± 14.18), the top-ranked model for the OTU domain was considered as a starting structure for Cezanne-2 and selected for subsequent refinement steps.

As a model for the diUb-bound Cez2 enzyme–substrate complex, we used a combination of the AF2 prediction and structural alignment. The FASTA sequences of both Cezanne-2 and two individual entities of Ub (UniProt ID: P0CG48, 76 amino acids each) were submitted to the multimer module of AF2.^49,50^ In all models, the distal Ub was correctly positioned close to Cez2, whereas the proximal Ub was positioned in an unrealistic binding mode far from Cez2 (Figure 1). Thus, the coordinates of the Cez2 OTU domain (average pLDDT score of 83.63 ± 16.59) and the distal Ub were retained. The distal Ub–Cez2 complex structure resembled the cocrystallized Cez OTU in complex with diUb (5LRV; Figure 1). Therefore, we used the 5LRV structure as a template to add the missing proximal Ub and complete the model of diUb–Cez2. The assignment of protonation states of amino acids (including Nε vs Nδ atoms of histidine residues) was verified by PROPKA and Epik, which are part of the Protein Preparation Wizard (Schrödinger 2022-2, LLC, New York, NY, 2022).^51,52^ To remove steric clashes, the initial structure was minimized in the absence of solvent using the steepest descent algorithm with a tolerance value of 10 kJ/mol. This was performed with the OpenMM program, using the CHARM36m force field.^53,54^

In the AF2 models of apo Cez2, the neutral state protonation state of the C210/H367 dyad and the large inter-residue distance of 0.58 nm were indicative of an “inactive” enzyme state.^41^ Structures with a zwitterionic C210^–^/H367^+^ dyad charge state were generated manually and then reminimized to remove steric clashes.

**Figure 2:**
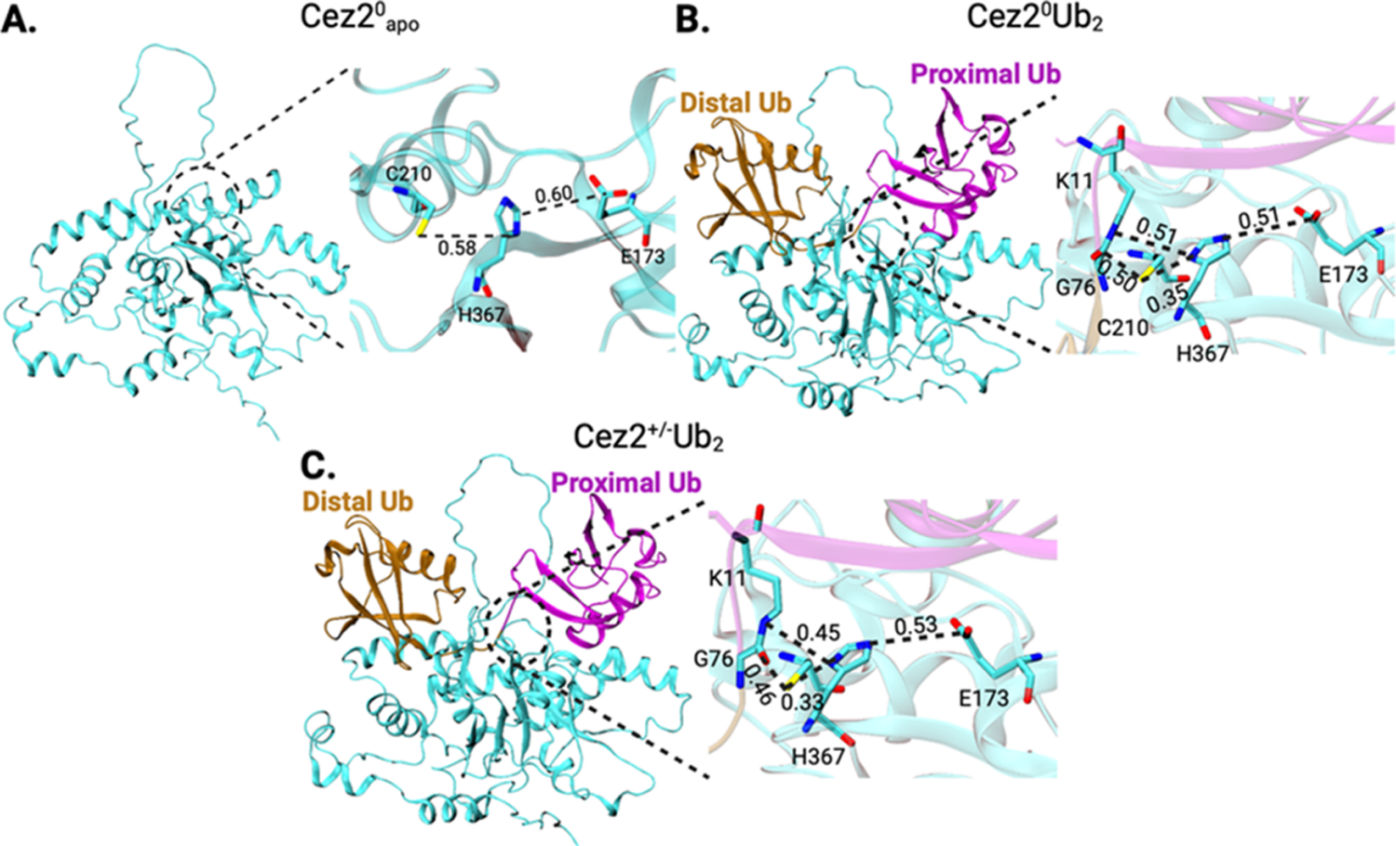
Structures of OTU domain of Cezanne-2 prior to MD simulations. (A) Catalytic center residues E173, C210, and H367 and the target G76-K11 isopeptide bond are shown in licorice representation. Relevant inter-residue distances are annotated in nm. (B) 3D structures of Cez2^0^Ub_2_ and (C) Cez2^+/–^Ub_2_, as well as distances between catalytic residues and the target G76-K11 isopeptide bond, which are depicted in licorice representation.

The energy minimized starting model structures for Cez2 comprise Cez2^0^apo, Cez2^0^Ub_2_, and Cez2^+/–^Ub_2_, and the C210(Sγ)···H367(Nδ) distances were 0.58, 0.35, and 0.33 nm, respectively (Figure 2). The E173(Cδ)···H367(Nε) distances were 0.60, 0.51, and 0.53 nm, respectively. Crystallization of DUB enzymes in complex with an unreactive diUb analogue is frequently done using a covalent activity-based probe (ABP), which leads to an incorrect substrate positioning at a large distance from the cysteine nucleophile.^20,32,55^ In the bacterial papain-like DUB RavD diUb crystal structure, for example, catalytic inter-residue distances are as large as 0.88 nm for the scissile bond to cysteine and 0.71 nm for the cysteine to histidine distance (PDB ID 6NJD). Also, the crystal structure of Cezanne-1 in the presence of an ABP was identified to correspond to a nonphysiological and catalytically inactive state and had to be refined by MD simulations.^32^

However, for cocrystallized human OTULIN, the substrate is in an aligned conformation with the scissile bond at 0.4 nm distance from the nucleophile (PDB ID 3ZNZ) and an inter-residue distance between active site cysteine and histidine of 0.34 nm. In the diUb-bound Cez2 protein–protein complex, the substrate isopeptide bond was found to be sufficiently close to the catalytic residue C210(Sγ) (0.50 nm for Cez2^0^Ub2 and 0.46 nm for Cez2^+/–^Ub2). These structural parameters suggest that the models corresponded to an enzyme–substrate complex with appropriate substrate positioning (see below).

The Cez2^0^Ub_2_ system was prepared from a snapshot of previous MD simulations of Cez^+/–^Ub_2_ in a prereactive configuration.^32^

### 2.2 Details of MD Simulations

All systems were subjected to the same MD protocol using the CHARMM36m force field and OpenMM program.^53,54^ The structures were solvated using the TIP3P water model and neutralized using the solvate and autoionize plugins of the Visual Molecular Dynamics (VMD) package.^56^ A final concentration of 0.15 M of NaCl was achieved to correspond to physiological conditions.^57^ Overall, the ions added were 59 Na^+^/45 Cl^–^ (for Cez2^0^apo), 60 Na^+^/48 Cl^–^ (for Cez2^0^Ub_2_ and Cez2^+/–^Ub_2_), and 72 Na^+^/57 Cl^–^ (for Cez^0^Ub_2_).

Initially, 5000 steps of steepest descent minimization were performed with a harmonic force constant of 500 kcal/mol nm^2^ applied to the backbone atoms (N–Cα–C–O). With this restraint still in place, the systems underwent relaxation in the NVT ensemble for 0.1 ns, followed by relaxation for 0.1 ns in the NPT ensemble. The temperature of 310 K was controlled by a Langevin integrator with a 1 ps^–1^ friction coefficient.^58^ A Monte Carlo barostat (coupled every 25 integration steps) was used to keep the pressure at 1 bar.^59^ Then, the restraints were removed, and 1-μs-long production runs were conducted in the NPT ensemble. Four independent MD replicas were performed for each system with different initial velocity distributions. Thus, the total accumulated simulation time reached 12 μs (Table 1). All MD simulations were performed under periodic boundary conditions with a time step of 2 fs. Nonbonded interactions were calculated using a switch and a cutoff distance of 1.0 and 1.2 nm, respectively. For long-range electrostatic interactions, particle mesh Ewald (PME) summation was applied with an error tolerance of 0.00001.^60,61^

**Table 1:** Overview of MD Simulations Performed in This Study

	system	protonation state of the Cys/His catalytic residues	nomenclature	number of replicates and simulation time per replicate	total simulation time (μs)
Cezanne-2	Ub-free (apo)	neutral	Cez2^0^apo	4 × 1 μs	4
	diUb-bound	neutral	Cez2^0^Ub_2_	4 × 1 μs	4
		zwitterionic	Cez2^+/–^Ub_2_	4 × 1 μs	4
Cezanne-1	diUb-bound	neutral	Cez^0^Ub_2_	4 × 1 μs	4

### 2.3 Analysis of MD Trajectories

The MD trajectories were analyzed using tools from GROMACS, the WaterBridgeAnalysis function of MDAnalysis, and in-house scripts.^62−64^ Data visualization was performed with the Seaborn and Matplotlib libraries.^65,66^ For histograms, the timeline data was divided into 50 equidistant bins to estimate the probability density. For 2D joint contour maps and cumulative density function (CDF) plots, kernel density estimation was utilized with the default settings of the Seaborn library. The trajectories were visualized with VMD.^56^ MD snapshots were rendered using Tachyon ray tracing.^67^ To evaluate the structural stability of the protein structure, MD trajectories were aligned with their respective initial structures, and the root-mean-square deviation (RMSD) of Cα atoms was calculated.

Standard quality measures were performed to ensure the stability of the MD simulations. The average Cα RMSDs of Cez2^0^apo, Cez2^0^Ub_2_, and Cez2^+/–^Ub_2_ from their respective initial structures were 0.50 ± 0.10, 0.47 ± 0.09, and 0.42 ± 0.06 nm, respectively. When residues adopting coil and turn structures were omitted, the Cα RMSDs decreased to 0.30 ± 0.07, 0.28 ± 0.05, and 0.30 ± 0.05 nm, respectively (Figure S1). Additionally, the Cα RMSD of regions with secondary structural elements was determined.

The Cez2–Ub interactions were calculated by measuring the minimum distances between heavy side-chain atoms of Cez2 and Ub residue pairs at 1 ns intervals via CONAN.^68^ Distances <0.55 nm were considered as a contact.

### 2.4 QM/MM Optimizations of MD Snapshots

Coordinates of substrate-bound Cez2 with a 0.7 nm water shell were extracted from selected MD snapshots (see below). The water molecules of the extracted systems were minimized at the MM level with the coordinates of the substrate-bound Cez2 complex fixed, using the CHARMM program (free version 42b1).^69^ Subsequently, the systems were optimized at the QM/MM level with the ChemShell package.^70,71^ TURBOMOLE (version 7.2) and DL_POLY (version 4.09) were used as QM and MM codes, respectively.^72,73^ The QM region consisted of relevant Cez2 residues (side chains of C210, E173, and H367), a fragment of diUb containing the reacting isopeptide group and involving a proper intrabackbone QM/MM boundary (side chain of K11, the entire G76 residue, and the carbonyl group (CO) of G75), and 0–2 water molecules bridging H367 and E173.^74^ The QM region has a total charge of −1. The hybrid functional B3LYP with a TZVP basis set was used.^75−80^ Optimizations were also performed with B3LYP-D3/def2-TZVP to check the effect of basis sets and to account for dispersion interactions by D3 corrections.^81^ The hybrid meta exchange–correlation functional M06-2X was used in single point calculations to confirm the hybrid DFT results.^82^ The rest of the system was in the MM region and treated with the CHARMM36 force field. All atoms within 1.1 nm of the QM region were unconstrained during optimizations (the “active” region), whereas all remaining atoms of the solvent, Cezanne-2, and diubiquitin were restrained.

The optimizations were performed using the DL-FIND optimizer module of ChemShell and hybrid delocalized internal coordinates (HDLC).^83,84^ An electrostatic embedding scheme with charge shift correction was used to compute the electrostatic interaction between the QM region and the surrounding partial charges of the MM region.^85,86^ Whereas the QM region has a charge of −1, the sum of formal charges of the entire system (QM, active, and frozen) was −12. Since the residues in the active region were different for each MD snapshot, the formal charges of the active region were between −6 and −2. No cutoff was applied for nonbonded interactions between the QM and MM regions.

**Figure 3:**
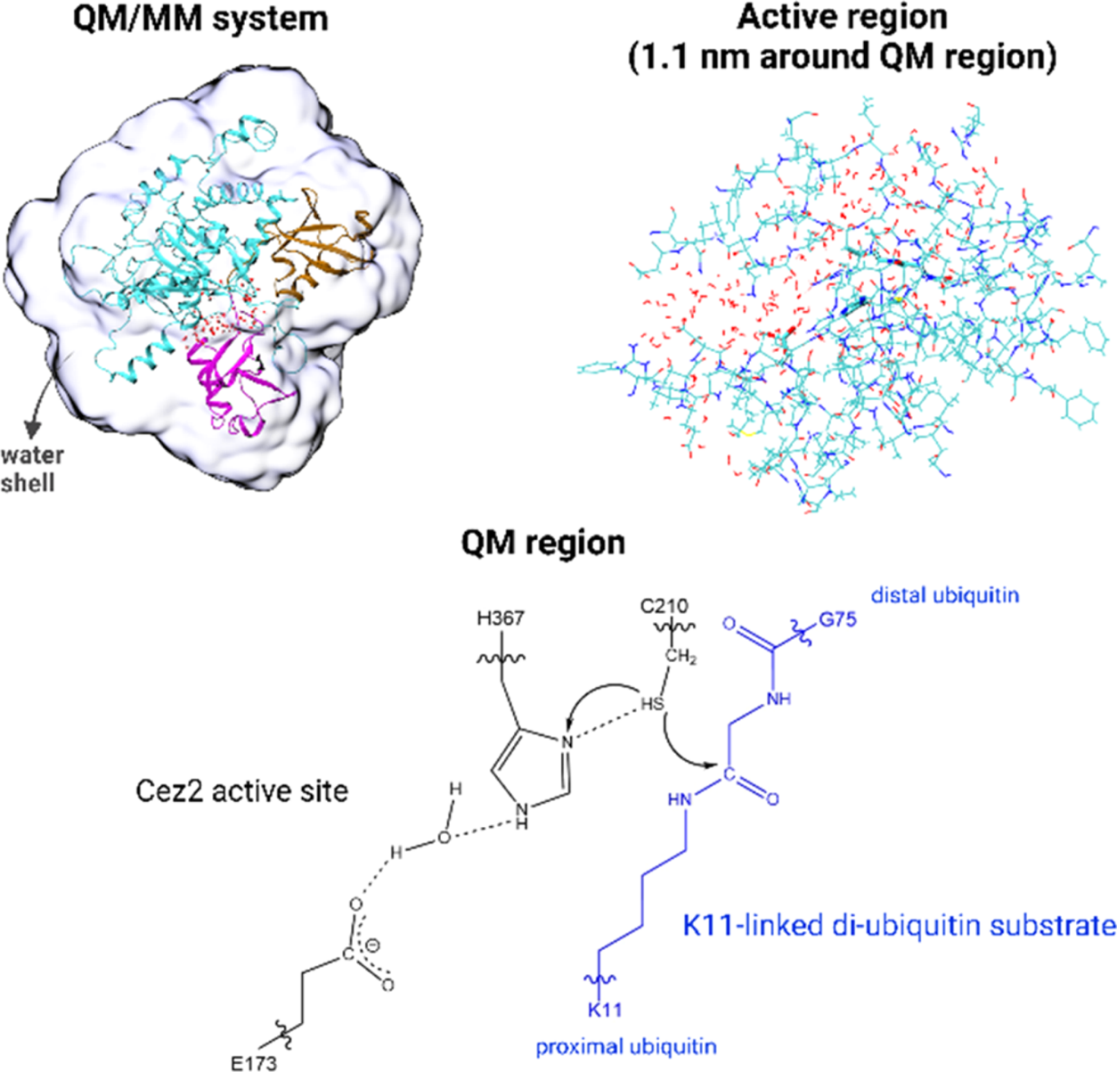
QM/MM system setup—Selected snapshots from Cez2^0^Ub_2_ and Cez2^+/–^Ub_2_ MD simulations with a 0.7 nm water shell were optimized at the QM/MM level. The QM region consisted of (i) the side chains of Cez2 residues C210, E173, and H367, (ii) the diUb isopeptide bond (the side chain of K11, G76, and the C=O group of G75), and (iii) (when occurring) water molecule(s) bridging H367 and E173. All atoms within 1.1 nm of the QM region were free to relax during optimization (active region). Residues outside the active region were treated at the MM level but were restrained.

Valencies at the covalent bonds crossing the QM/MM boundary were saturated using hydrogen link atoms.^87^ The chosen QM/MM setup is similar to previous studies reported in the literature^74,75,88,89^ (Figure 3).

## 3 Results and Discussion

### 3.1 Equilibrium of Neutral and Zwitterionic Cezanne-2 Charge States Shifts upon Substrate Binding

Cez2 is a cysteine protease with a tentative cysteine–histidine catalytic dyad or a cysteine/histidine/glutamate triad. Structural differences between the active site residues in the free and diUb-bound states can be used to shed light on the state of activation of DUBs.^55^

The possibility of a substrate-assisted enzymatic activation mechanism can be investigated by monitoring relevant structural arrangements within the catalytic center between apo form and the enzyme–substrate complex.^90^ For cysteine proteases, the enzymatic cleavage of the substrate isopeptide bond is preceded by an *in situ* generation of the cysteinate nucleophile, which is assisted by the nearby histidine and gives a zwitterionic Cys^–^/His^+^ charge state. The DUB proteins OTUB1 and OTUB2 differ in their states of activation of the active site prior to substrate binding. Whereas OTUB1 achieves a prereactive conformation only upon ubiquitin binding and then subsequently forms the charge-separated state, the catalytic residues in OTUB2 are in a catalytically productive configuration even in the absence of ubiquitin binding.^43^ For Cez, even in the absence of the diubiquitin substrate, an equilibrium between the inactive and active states of the catalytic dyad was seen for the apoenzyme.^32^

In order to investigate the state of activation of Cez2 in the absence and presence of diubiquitin, the inter-residue distances of the active site C210(Sγ) and H367(Nδ), and H367(Nε) and glutamate E173(Cδ) were monitored in the apo and diubiquitin bound states of Cez2. Figure S2 shows the probability density distributions of C210(Sγ)···H367(Nδ) and E173(Cδ)···H367(Nε) distances in the different states of activation of Cez2.

**Figure 4:**
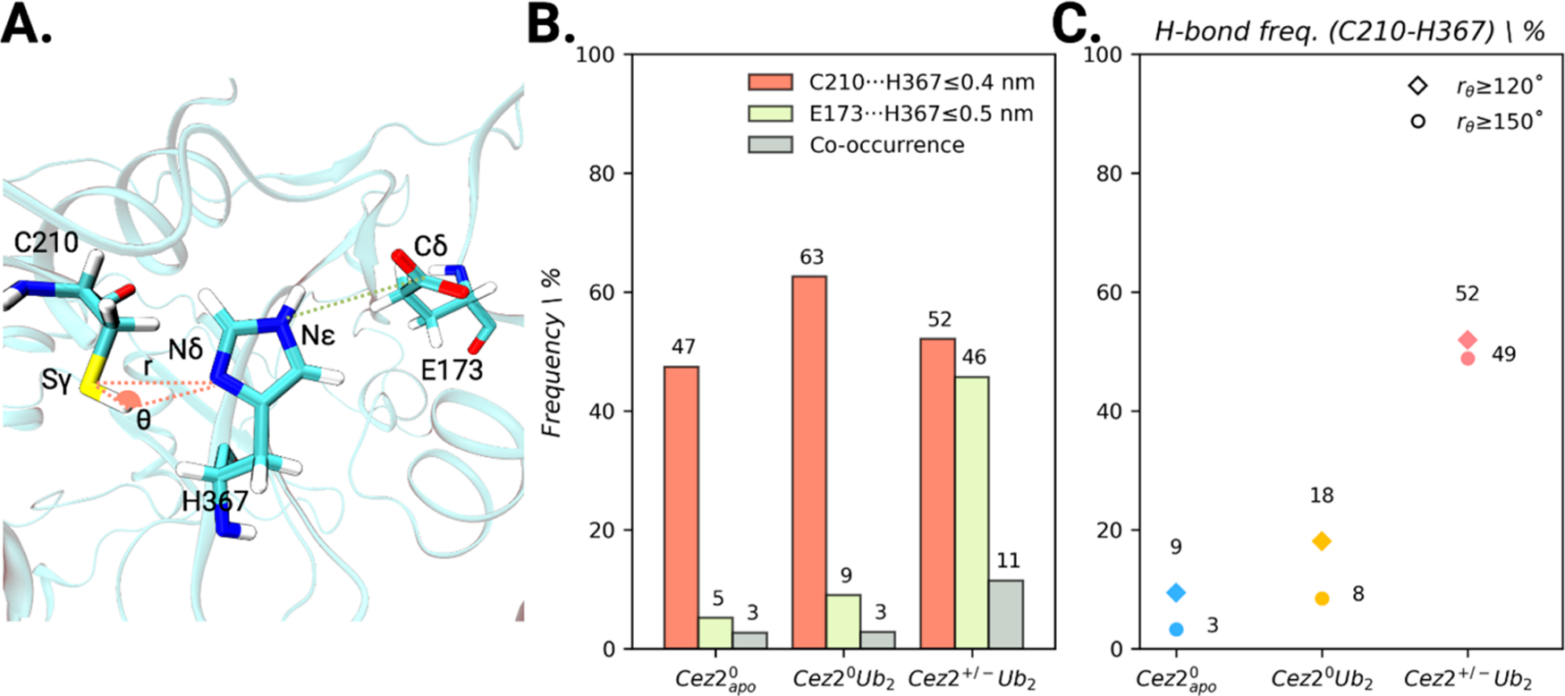
Monitoring of hydrogen bond formation between active site residues. (A) Definition of structural criteria for hydrogen bond formation between active site residues: the distance *r* between Sγ and Nδ and the angle **θ** (Sγ-H···Nδ). (B) Probability of hydrogen bonding. Distances C210(Sγ)···H367(Nδ) ≤ 0.4 nm (orange bar), E173(Cδ)···H367(Nε) ≤ 0.5 nm (lime bar), and both criteria simultaneously (gray bar). (C) Number of MD frames with distance *r*(C210(Sγ)···H367(Nδ)) ≤ 0.4 nm and angle **θ**(Sγ-H···Nδ) ≥ 120° (diamond) or ≥150° (circle).

The heavy atom distance *r* was used to identify the formation of a hydrogen bond between the thiol group of cysteine and the Nδ atom of the histidine (see Figure 4A). Upon substrate binding, the number of MD frames reaching a cysteine–histidine hydrogen bond distance *r* ≤ 0.4 nm was 63 and 52% of the simulation time for Cez2^0^Ub_2_ and Cez2^+/–^Ub_2_, respectively (Figure 4B). Cez2^0^apo exhibited short C210(Sγ)···H367(Nδ) inter-residue distances for approximately 47% of the simulation time and thus behaves similarly (Figure 4B).

A distance criterion alone is not sufficient to describe the formation of a hydrogen bond, but also the angle **θ** between hydrogen bond donor (X) and acceptor (Y) atoms needs to be considered (see Figure 4A).^91^ The X–H···Y angle should preferably be above 110° for a hydrogen bond, depending on the system. For Cez2^0^apo, even in the absence of substrate binding, 9 and 3% of the short interaction distance snapshots were prone to form hydrogen bonds when criteria of θ ≥ 120 or 150° are applied, respectively (Figure 4C). Thus, the formation of the zwitterionic charge state is also feasible in the absence of diubiquitin binding. An equilibrium between neutral and zwitterionic charge states of the active site residues cysteine and histidine can thus be observed.

Upon consideration of the angle of hydrogen bonding, 18 and 52% for Cez2^0^Ub_2_ and Cez2^+/–^Ub_2_ (θ ≥ 120°) and 8 and 49% (θ ≥ 150°) of the short distance MD frames were prone to form a hydrogen bond (Figure 4C). These results indicate that substrate binding increases the number of configurations, facilitating the C210–H367 proton transfer, formation of the zwitterionic charge state, and activation of the enzyme. Therefore, substrate binding to Cezanne-2 promotes the formation of short cysteine–histidine distances (63% of simulation frames) and enhances the formation of the zwitterionic charged active site, thereby activating the catalytic dyad. For Cez, substrate binding also shifted the equilibrium between the neutral and zwitterionic charge states toward the latter and promoted OTU activation.^32^

### 3.2 Third Residue in the Active Site of Cezanne-2 Is Not Directly Involved in Isopeptide Bond Cleavage

A recent review provides insight into the variety in DUB catalytic mechanisms by comparing structural parameters of crystallized DUBs in the absence and presence of the substrate.^92^ DUB cysteine proteases may employ either a dyad of cysteine and histidine residues or involve a critical third residue as well. The role of this third residue can be indirect by stabilization of the charge-separated state of the DUB (for example, Asp),^43^ it may be directly involved in the reaction mechanism (for example, Ser or Gln),^20^ or support substrate recognition and binding.^32^ In order to elucidate the possible formation of a catalytic triad or the involvement of the third active site residue E173 in the reaction mechanism of Cezanne-2, the inter-residue distance between E173(Cδ) and H367(Nε) was monitored (Figure 4B). A distance threshold of ≤0.5 nm was used as an indicator for a direct interaction. E173 approaches H367 in only 5 and 9% of the MD frames in the apo and neutral diUb states of Cez2. The formation of a catalytic triad E173–C210–H367 occurs for only 3% (in Cez2, Cez2^0^Ub_2_) of the simulation time in the neutral and 11% in the zwitterionic Cez2^+/–^Ub_2_ state. Apparently, the third residue in the active site of Cez2 is not directly involved in the stabilization of the catalytic residues. For Cez2^+/–^Ub_2_, interactions between either Cys^–^/His^+^ (52%) or His^+^/Glu^–^(46%) seem to be almost equally probable but occur only very rarely simultaneously. Also, for Cez, the approach of C194 to H358 was found not to be correlated with a close E157···H358 interaction. Residue E157 was not directly promoting the formation of a zwitterionic charge state in the active site. Also, for Cezanne-1, the corresponding residues E157(Oε1)···H358(Nδ) were predominantly at large distances and not below 0.75 nm. This provided evidence of the presence of a catalytic dyad instead of a triad, which could only be suggested before.^29^

Upon substrate binding, distances ≤0.5 nm between residues E173 and His367 can be seen for 46% of the simulation time for Cez2^+/-^Ub_2_. Meanwhile, distances ≤0.4 nm between residues C210 and H367 can be seen for 52% of the simulation time. Thus, we aim to identify whether E173 interaction with the catalytic center is possibly mediated by one or several water molecules rather than forming a direct hydrogen bond with H367.

### 3.3 Strictly Conserved Water Bridge Stabilizes the Orientation of Active Site Residues

**Figure 5:**
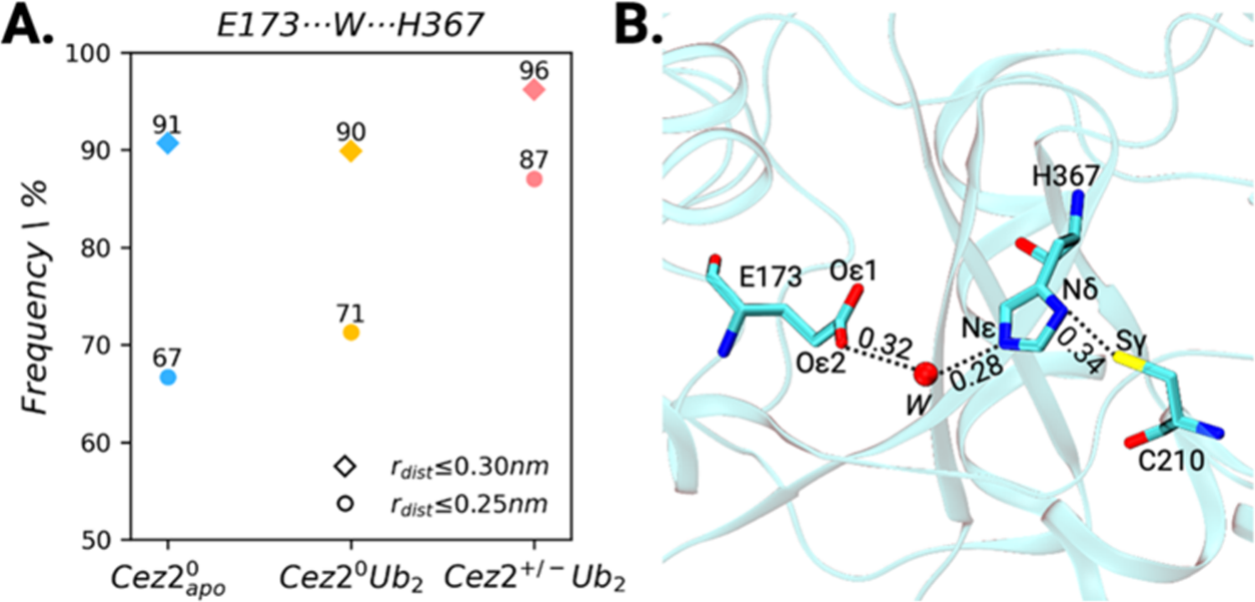
Water-mediated interaction between E173 and H367 in Cezanne-2. (A) Frequency of MD frames with one to three water molecules bridging E173 to H367, using a cutoff distance *r*_dist_ ≤ 0.25 nm (circle) and ≤0.30 nm (diamond). (B) Representative MD snapshot of Cez2^0^apo showing a conserved water molecule between E173 and H367, along with the formation of a hydrogen bond between C210 and H367, enabling proton transfer. Distances are labeled in nm.

In order to identify a further interaction partner of the Cezanne-2 E173 residue, the possibility of conserved water molecules in the vicinity of the active site was investigated. As a threshold to identify water molecules close to the Cez2 active site residues, distances between E173(Oε) and a water oxygen atom and the water oxygen atom and H367(Nε) of ≤0.30 nm were defined. Figure 5A shows that at least one water molecule was localized for 91, 90, and 96% of the simulation time in the Cez2^0^apo and the diUb-bound Cez2^0^Ub_2_ and Cez2^+/–^Ub_2_ states. When the cutoff distance was reduced to 0.25 nm, at least one water molecule was detected in 67, 71, and 87% of the simulation time for Cez2^0^apo, Cez2^0^Ub_2_, and Cez2^+/–^Ub_2_.

The presence of the conserved water molecule thus seems not to be critically dependent on substrate binding vs absence and the charge states of the cysteine and histidine residues. It bridges the active site residues histidine and glutamate in all states of Cezanne-2. We can thus state that the third residue, E173, is not directly involved in the catalytic mechanism of Cezanne-2 but that its water-mediated hydrogen bonding with H367 may enhance the basicity of the latter.

A QM charge analysis using natural populations analysis (NPA) was done to obtain natural atomic charges (NACs) for residues of the reaction network C210–H367–H2O–E173 in the prereactive configuration D4 of the Cez2^0^Ub_2_ protein–protein complex (see below). For three QM/MM-optimized MD snapshots, the atomic charges of (i) the QM region polarized by the MM point charges (QM/MM, Figure 3), (ii) the QM region in the absence of MM (QM), (iii) the QM region with removed water (QM1), and (iv) the QM region with removed water and removed residue E173 (QM2). The results are given in Table 2.

**Table 2:** Calculated Natural Atomic Charges of the C210 and H367 Proton Donor and Acceptor Atoms of Cez2^0^Ub_2_ at the Prereactive Configuration D4

	snapshot 1^a^				snapshot 2				snapshot 3			
atom	QM/MM	QM	QM1	QM2	QM/MM	QM	QM1	QM2	QM/MM	QM	QM1	QM2
C210:Sγ	–0.16	–0.13	–0.12	–0.10	–0.16	–0.12	–0.11	–0.09	–0.18	–0.14	–0.13	–0.11
C210:Hγ	0.18	0.18	0.17	0.16	0.19	0.17	0.17	0.16	0.19	0.18	0.18	0.17
H367:Nδ	–0.52	–0.51	–0.50	–0.48	–0.55	–0.51	–0.50	–0.47	–0.53	–0.51	–0.49	–0.47
H367:Nε	–0.50	–0.50	–0.50	–0.50	–0.49	–0.50	–0.49	–0.50	–0.49	–0.50	–0.49	–0.50
H367:Hε	0.46	0.46	0.43	0.42	0.45	0.46	0.44	0.41	0.46	0.46	0.44	0.42

a Numbering matches those in Table S1.

In the QM/MM calculations, the charge of cysteine Sγ is higher than in the mere QM cluster calculations. Furthermore, the positive charge of the C210:Hγ atom is higher in the presence of the bridging water molecule, pointing to a higher acidity. The water-mediated interaction between H357 and E173 (compare QM, QM1, and QM2) is thus also polarizing the remote cysteine residue, facilitating deprotonation and increasing its nucleophilicity. In the presence of the bridging water molecule, the H367:Nδ atom charge is between −0.51 to −0.55 and −0.47 to −0.48 when it is absent. Thus, the presence of a water molecule increases the polarization of the histidine nitrogen atom Nδ and its basicity and favors protonation of Nδ by the Cys210:SγH group.

**Figure 6:**
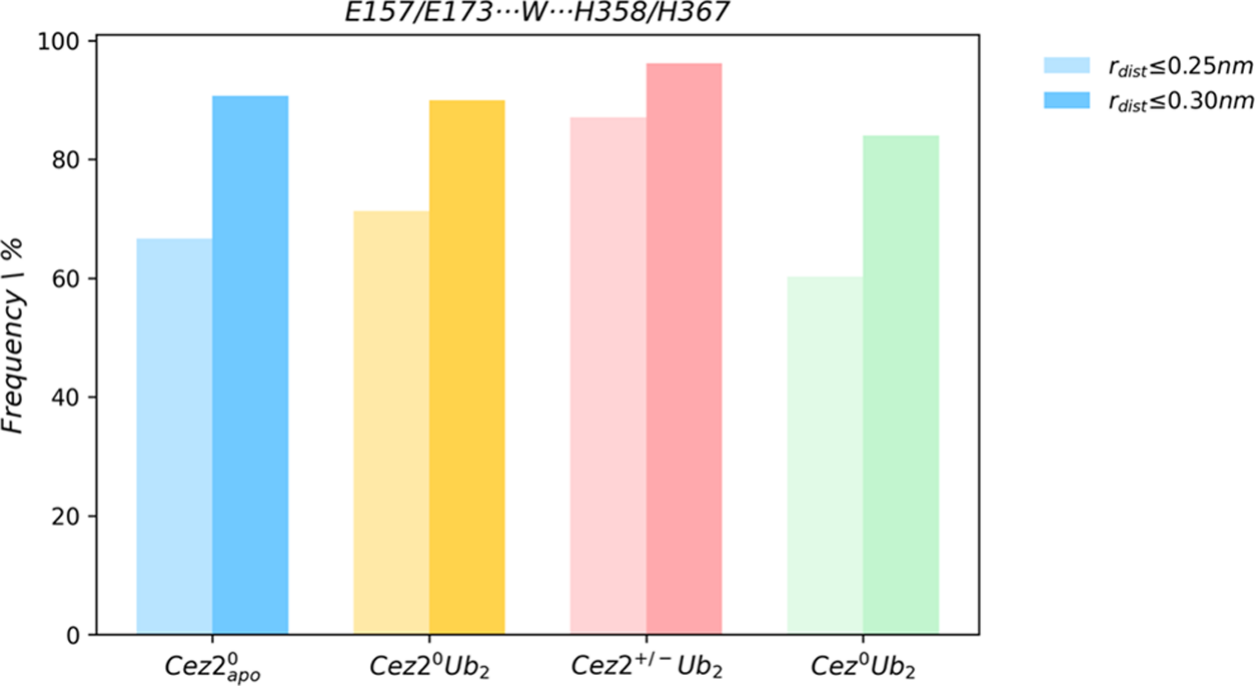
Comparison of water-mediated interactions in Cezanne-1 and Cezanne-2. Frequency of MD frames with at least one water molecule (up to 3 water molecules) bridging E157/E173 to H358/H367 when using a cutoff distance *r*_dist_ ≤ 0.25 nm (faded) and ≤0.30 nm.

For Cezanne-1, a water molecule was localized for 60 and 84% of the simulation time in Cez^0^Ub_2_ when the cutoff distance was set to 0.25 and 0.30 nm, respectively. Given the predominant large E157···H358 distances detected in Cez and the conserved water molecule (Figure 6), residue E157 in combination with the bridging water molecule may also increase the basicity of H358.

For the OTUB1 DUB in its zwitterionic, substrate-bound state, a strong charge interaction between catalytic site residues H265^+^ and D267^–^ was persistent throughout the simulations (at a distance of 0.27 ± 0.01 nm). For OTUB2, however, the inter-residue distance between His224^+^ and the nearby neutral Asn226 was significantly larger (0.75 ± 0.18 nm), and a water molecule was identified to occupy the position between these residues, which was not resolved in the crystal structure.^43^

**Figure 7:**
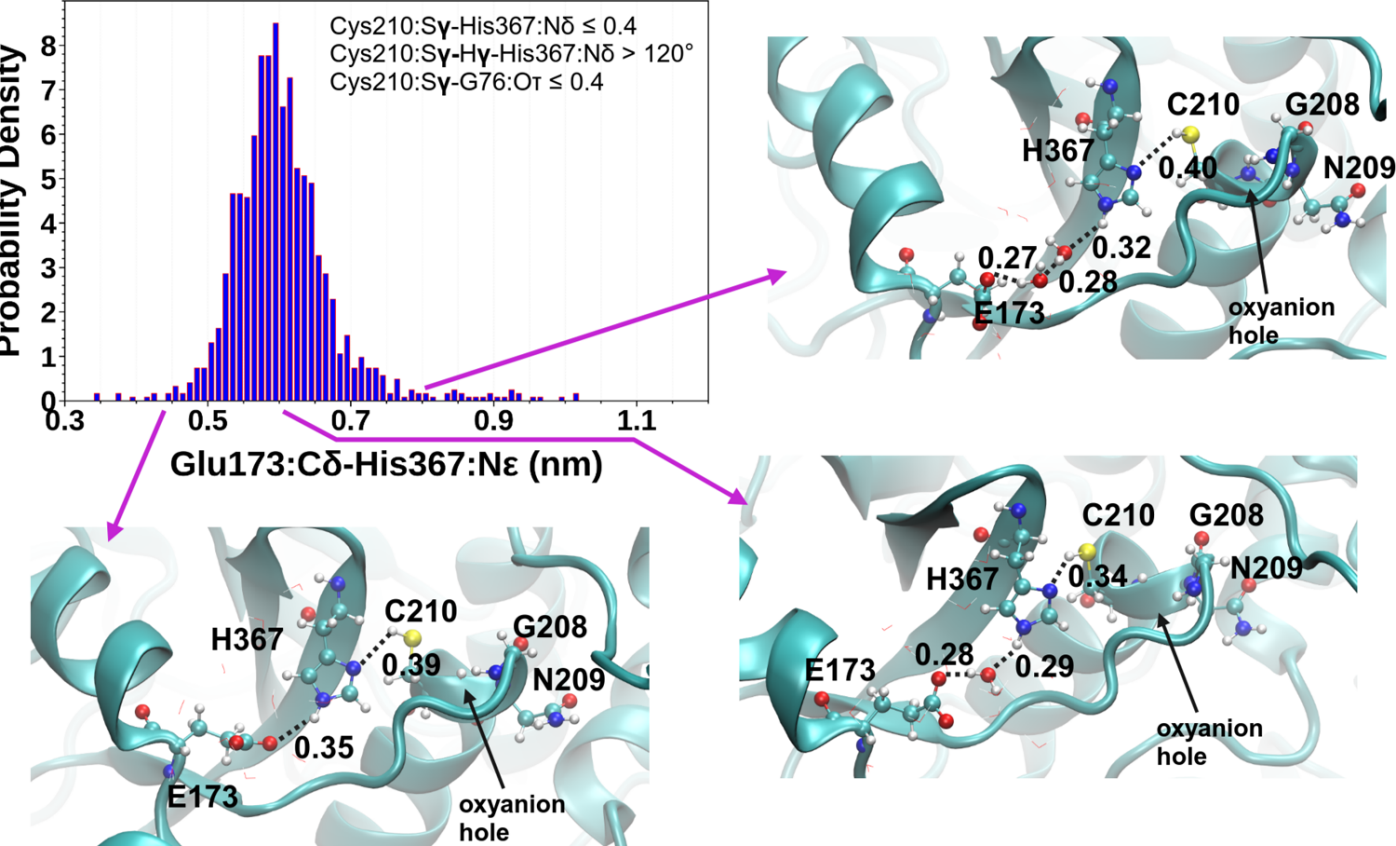
Probability density plot of the E367:Cδ-H367:Nε interatomic distances for catalytically competent configurations of the Cez2^0^Ub_2_ enzyme–substrate complex, along with representative MD snapshots (distances in nm).

Figure 7 shows the probability density distribution of E173···H367 distances in the enzyme–substrate Cez2^0^Ub_2_ complex in a prereactive configuration. Short distances of about 0.35 nm rarely occur and correspond to a direct hydrogen bond interaction between these residues. Large distances of 0.8 nm are also rarely sampled and refer to E173 and two bridging water molecules mediating the interaction with H367. The most frequently sampled inter-residue distance of 0.52–0.68 nm is characteristic for one water molecule bridging residues E173 and H367.

### 3.4 Dual Role of the Third Active Site Residue in Cezanne-2

For Cezanne-1, diubiquitin substrate binding boosted the catalytic competency of the OTU. Mutations of the third glutamate residue, E157, led to reduced or even complete absence of proteolytic activity.^29^ The third residue was identified to be critically relevant for enzymatic catalysis, although not directly involved in the cleavage of the isopeptide bond. The electrostatic interaction between the catalytic center residue E157 and a nearby K33 of the proximal Ub was responsible for substrate recognition and was present for 60% of the simulation time.^32^

**Figure 8:**
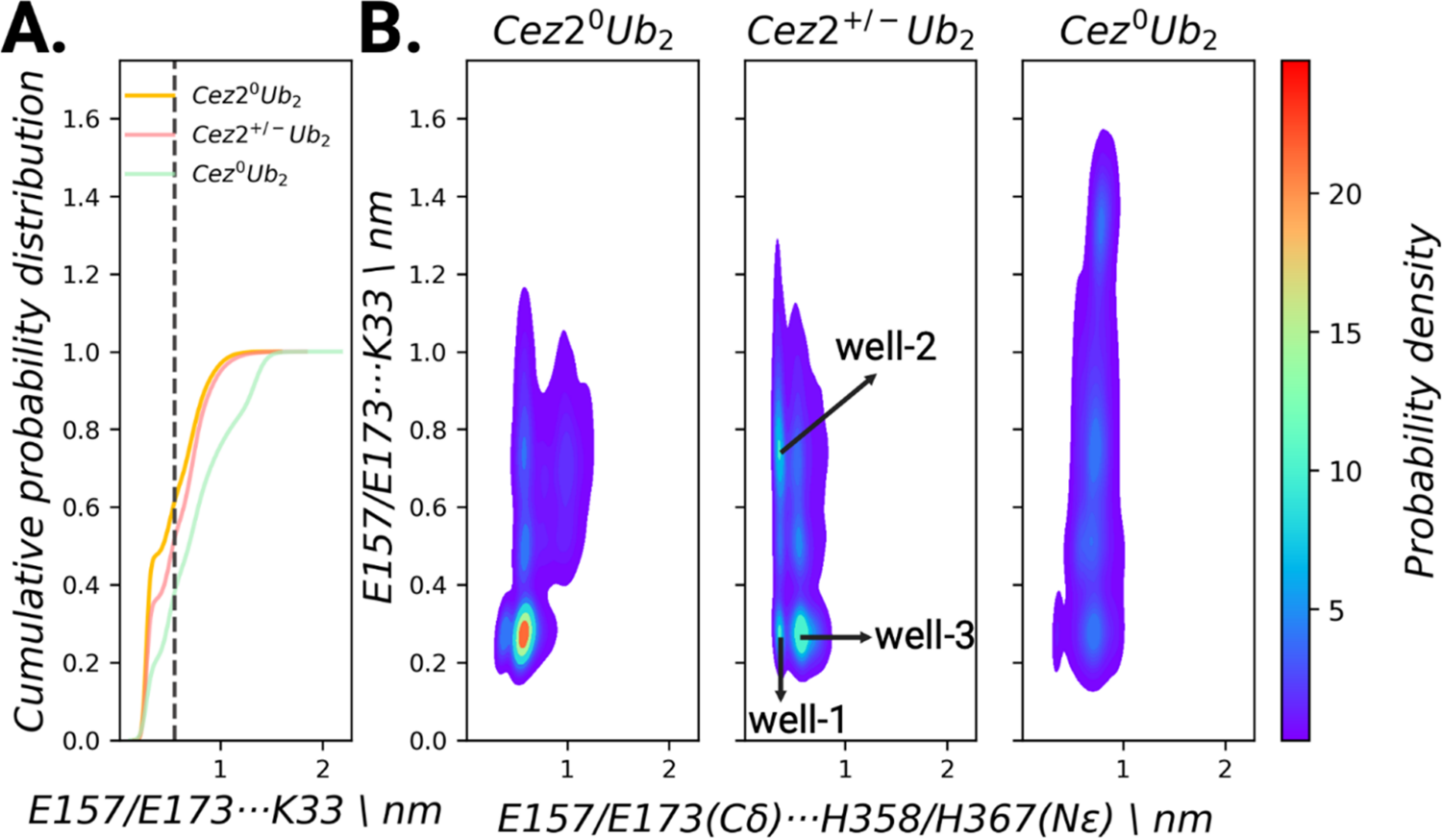
The dual role of E173 from Cez2 in substrate recognition of Ub and stabilization of the catalytic site residue H367. (A) Monitoring of Cez2 interactions with proximal Ub in neutral and zwitterionic OTU-diUb enzyme–substrate complexes. The dashed line represents a cutoff distance of 0.55 nm. (B) 2D joint probability contour maps for Cez2 E173(Cγ)···H367(Nε) and proximal Ub E173(Oε1/Oε2)···K33(Nζ) interactions in neutral and zwitterionic OTU-diUb enzyme–substrate complexes compared to Cez^0^Ub_2_.

Cezanne-2 displays the same selectivity toward K11-polyUb,^14^ and we investigated the interaction between the third residue of the OTU active site (E173) and a positively charged lysine residue (K33) of the substrate proximal Ub. The distance between E173(Oε1/Oε2) and K33(Nζ) was monitored for the MD trajectories of neutral and zwitterionic OTU-diUb complexes. The time evolution of relevant distances E-H and E-K can be found in Figure S7. For the charge states Cez2^0^Ub_2_ and Cez2^+/–^Ub_2_, this distance was between 0.25 to 1.50 nm and 0.25 to 1.74 nm, respectively. In over 50% of MD frames for both neutral and charged diUb-bound Cez2, the E173(Oε1/Oε2)···K33(Nζ) distance was shorter than 0.55 nm, indicating a long-living electrostatic interaction between these residues in both states (Figure 8A, where the dashed line represents the cutoff distance of 0.55 nm). As observed for Cez, recognition and binding of the K11-linked proximal ubiquitin shifts the equilibrium of the catalytic residues toward the zwitterionic charge state. The persistent interaction between E173 of Cez2 and K33 of the substrate suggests that this is an important factor to stabilize the proximal Ub–Cez2 binding.

Thus, for the third catalytic site residue E173, a similar dual functional role as for Cez can be suggested: it is critical for the recognition and stabilization of proximal Ub binding to Cez2 (for Cez2^0^Ub_2_: 61% of the simulation time, for Cez2^+/–^Ub_2_: 52%) and the stabilization of the Cys/His dyad by a water-mediated interaction with H367 as E173 makes a water bridge for ≥90% of the simulation time. The co-occurrence of the water bridge between E173 and H367 and the electrostatic interaction between Cezanne-2 E173 and substrate K33 was detected for 59 and 50% of the simulation time for Cez2^0^Ub_2_ and Cez2^+/–^Ub_2_, respectively, when *r*_dist_ was set to 0.30 nm. Thus, the charge state of the active site residues does not affect the binding of the proximal ubiquitin. For Cez, the co-occurrence of the water-mediated stabilization of the dyad and proximal ubiquitin was lower (31%) for Cez^0^Ub_2_, which indicates a less strong binding of the proximal Ub when the active site is in its neutral state.

2D contour maps of the E173/H367 and E173/K33 distances show the interaction in both charge states of the catalytic dyad (Figure 8B). When the active site is in a neutral charge state, the interaction has only one highly populated region, corresponding to a local minimum at an E173/K33 distance of ∼0.27 nm and E173/H367 of ∼0.57 nm. This is in agreement with the water-mediated interaction between glutamate and histidine and the electrostatic interactions between Cez2 and K33 from the proximal Ub. However, in the zwitterionic Cez2 protein–protein complex, several local minima can be identified on the 2D joint probability contour map. In these wells, the same binding mode between E173(Cγ)···H367(Nε)/E173···K33 as in the neutral Cez2 state can be seen (well-3). However, at distances of 0.35/0.75 nm for E173(Cγ)···H367(Nε)/E173···K33 (well-2), the water-mediated interaction with H367 is present, but the electrostatic interaction with the substrate is absent. There is also a shallow minimum indicative for a simultaneous direct interaction between E173(Cγ)···H367(Nε)/E173···K33 at 0.36/0.27 nm (well-1). This corresponds to a direct salt-bridge interaction between E173^–^/H367^+^ that is not mediated by a water bridge.

For neutral Cez in complex with diUb, also two local minima could be observed with almost equal distribution: one corresponding to a direct salt-bridge interaction (at 0.25 nm; well-1) between E157 and K33 and a second at 0.5 nm, which is indicative of the absence of substrate fixation but maintaining the catalytic dyad stabilization (shown by short C194/H358 distances; well-2).

The analysis of the zwitterionic charge state of Cez2 in complex with diUb shows the dual role of E173: there is an almost equal distribution between E173 enabling substrate binding and stabilization of the orientation of the catalytic site to promote its protease activity. Compared to that of Cezanne-1, the charge state of the catalytic center has no significant impact on the electrostatic interaction between E173 and H367. On the other hand, in Cez, the electrostatic interaction between E157 and the proximal ubiquitin residue K33 increases from 39 to 60% of the simulation time upon formation of the zwitterionic charge state. Apparently, the binding of the proximal ubiquitin is enhanced only upon the formation of the zwitterionic charge state in Cez.

### 3.5 Identification of Prereactive Configurations of Cezanne-2 in Complex with Diubiquitin

In the absence of a substrate, Cez2 maintains an equilibrium between the neutral and zwitterionic charge states of the catalytic residues C210 and H367. Upon diUb substrate binding, however, this equilibrium shifts toward the zwitterionic and prereactive states with an aligned active site.

**Scheme 1:**
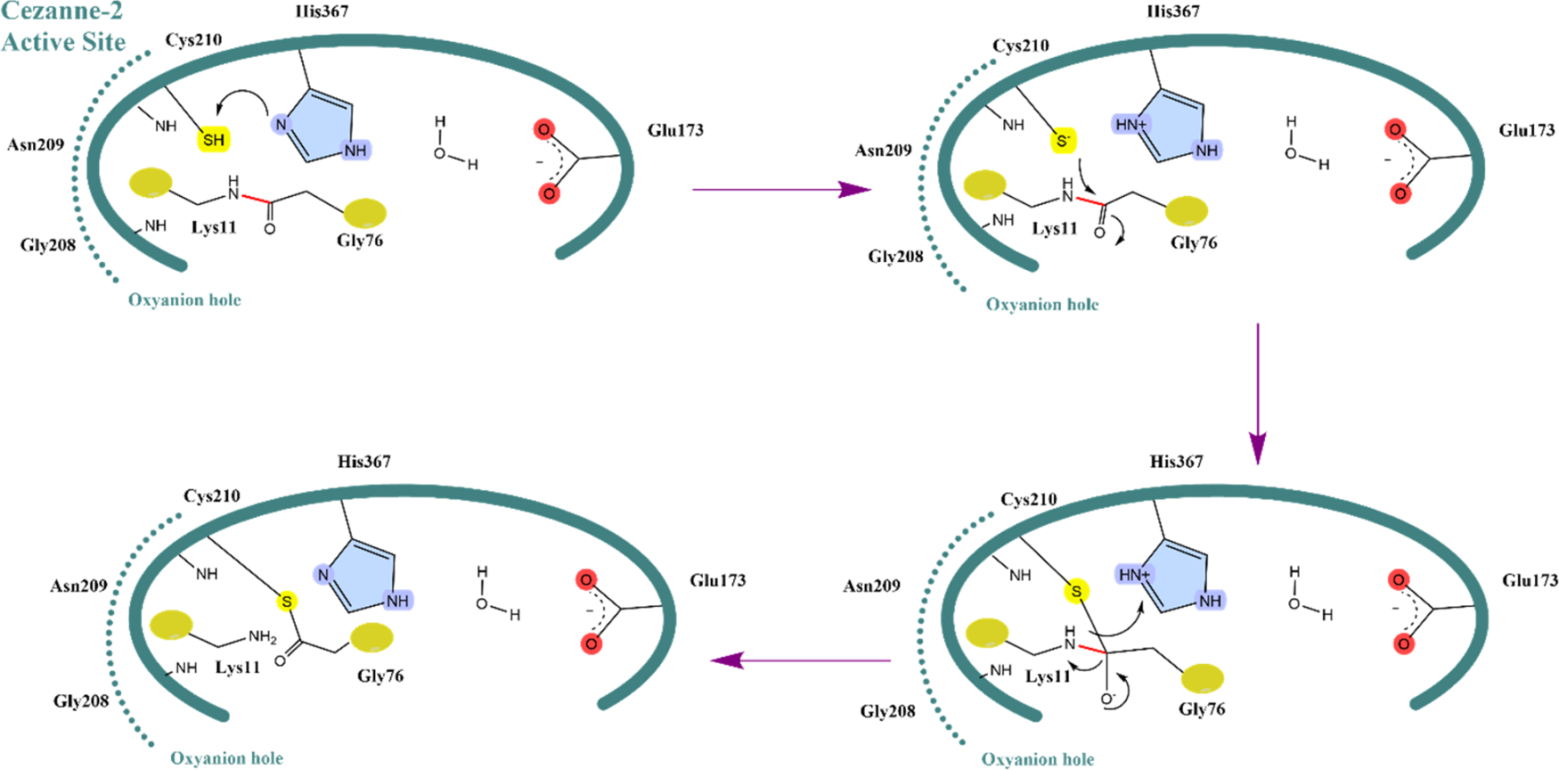
Suggested Mechanism for the Proteolytic Reaction of Cez2 toward K11-Linked Diubiquitin Formation of the zwitterionic charge state of the catalytic residues Cys210 and His367 requires a proton transfer. The water-mediated interaction between His367 and Glu173 helps to stabilize the prereactive orientation. The thiolate nucleophile approaches the carbonyl carbon electrophile of the isopeptide bond (in red) and forms a covalent thioester with the substrate. The negative charge of this tetrahedral intermediate is stabilized by hydrogen bonds from residues of the “oxyanion hole.” Cleavage of the isopeptide bonds gives the acyl-enzyme intermediate from which the cysteine and glycine amino acids are recovered due to hydrolysis (not shown).

In order to identify catalytically competent Cezanne-2 diUb complex structures, different geometrical criteria were applied to filter the MD trajectories and identify prereactive/reactive configurations considering commonly accepted reaction mechanisms of cysteine proteases (Scheme 1 and Table 2).

**Figure 9:**
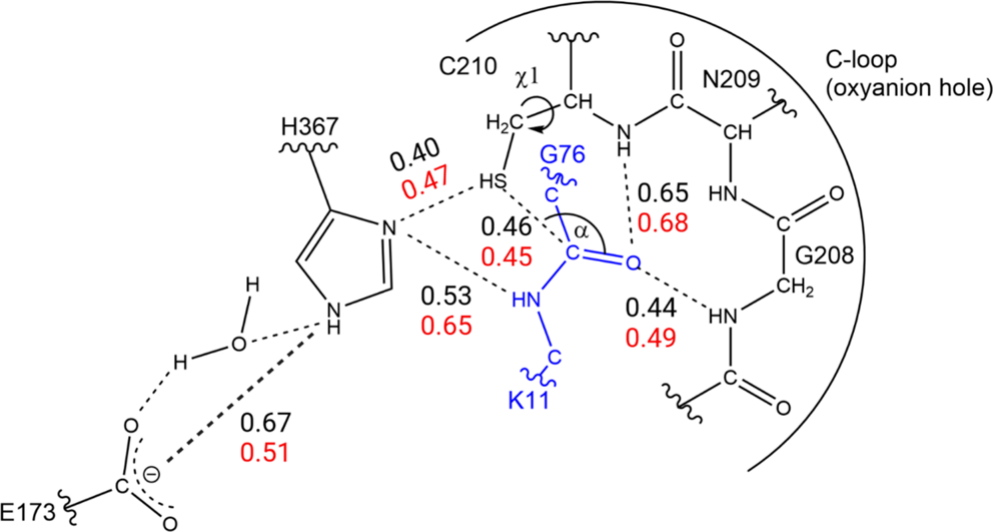
Schematic view of the Cez2^0^Ub2 complex showing relevant interactions (interatomic distances as dashed lines), Bürgi–Dunitz angle (α) for the nucleophilic attack, and C210 side-chain dihedral angle orientation (χ1) to assess the formation of prereactive configurations for K11-Gly76 isopeptide bond hydrolysis by Cez2. Average distances are in nanometers for the neutral state (black) and the zwitterionic (red) state of the dyad. See Table 3 and text for more details.

The nucleophilic attack of the C210 thiolate to the isopeptide bond of G76-K11 requires not only a short inter-residue distance but also an appropriate orientation of the nucleophile. The Bürgi–Dunitz angle is a descriptor of the geometry of a nucleophile attack on a sp^2^-hybridized carbonyl center, here an isopeptide bond, and usually between 107 and 109.5° (Figure 9).^93^

To evaluate the possibility of a nucleophilic attack by the thiolate C210(Sγ) atom on the carbonyl carbon of the G76-K11 isopeptide bond, we considered (i) the C210(Sγ)···G76(C) interatomic distance and (ii) the corresponding C210(Sγ)···G76(C)···G76(Oτ) angle α as appropriate descriptors (Figure 9 and Table 3), among others. Since classic force fields are parametrized to describe equilibrium bond distances, angles, and dihedral angles and the Bürgi–Dunitz angle describes the quantum chemical interaction between the *n* orbital of the nucleophile with the π* orbital of the polarized carbonyl moiety (as a result of this partial charge transfer, the carbonyl carbon is pulled out of the carbonyl plane), a BD angle of 107 ± 20° was used as a threshold. This cutoff value was also recently used to identify prereactive states for the USP7 DUB.^94^

**Table 3:** Geometrical Descriptors for the Identification of Potential Productive Configurations for Cez2^0^Ub_2_

criteria	geometrical descriptor and threshold	associated structural property
I	C210(Sγ)···G76(C) distance ≤0.4 nm	nucleophilic attack distance
	C210(Sγ)···G76(C)···G76(Oτ) angle 107 ± 20°	nucleophilic attack Bürgi–Dunitz angle
II	C210(Sγ)···H367(Nδ) distance ≤0.4 nm	hydrogen bond between C210 and H367
	C210(Sγ)···H367(Nδ) < C210(Sγ)···H367(Nε)	proton transfer with Nδ as proton acceptor^a^
III	H367(Nδ)···K11(Nζ) distance ≤0.6 nm	hydrogen bond between H367 and K11
IV	S302(Oγ)···G76(Oτ) distance ≤0.4 nm	hydrogen bond stabilization of isopeptide C=O by S302
V	S302(Oγ)···G76(Oτ) distance >0.4 nm	absence of hydrogen bond between isopeptide C=O and S302
VI	χ1(C210(N)···C210(Cα)···C210(Cβ)···C210(Sγ)) < 0°	side-chain orientation of C210 with respect to the C-loop
VII	χ1(C210(N)···C210(Cα)···C210(Cβ)···C210(Sγ)) > 0°	

a Considering H367:Nε as a proton acceptor was unsuccessful.

A set of configurations are identified, which are referred to as B, C, and D3–D6, which fulfill several criteria for a successful isopeptide bond cleavage. Table 4 provides the number of identified prereactive configurations in Cez2 and Cez when in complex with the diubiquitin substrate.

**Table 4:** Number of MD Snapshots from Cez2^0^Ub_2_ and Cez2^+/–^Ub_2_ Fulfilling Structural Criteria for a Reactive Configuration Out of a Total Number of 40,000 MD Frames

		Cez2^0^Ub_2_		Cez2^+/–^Ub_2_		Cez^0^Ub_2_	
criteria fulfilled	configuration	no. of frames	% freq	no. of frames	% freq	no. of frames	% freq
I, II	B	543	1.36	313	0.78	776	1.94
I, II, III	C	540	1.35	255	0.64	697	1.74
I, II, III, IV, VII	D3	2	0.01	18	0.045	40	0.01
I, II, III, V, VII	D4	154	0.39	53	0.13	372	0.93
I, II, III, IV, VI	D5	187	0.48	97	0.24	78	0.19
I, II, III, V, VI	D6	197	0.49	87	0.22	207	0.52

Configuration B refers to those MD frames where the catalytic center and the diUb isopeptide bond are structurally arranged to enable both the C210–H367 proton transfer and C210-isopeptide nucleophilic attack (by distance and orientational criteria). Configurations C are MD frames that, in addition to the above criteria, also facilitate the H367–K11 proton transfer that is expected to occur at a later stage of the reaction.

**Figure 10:**
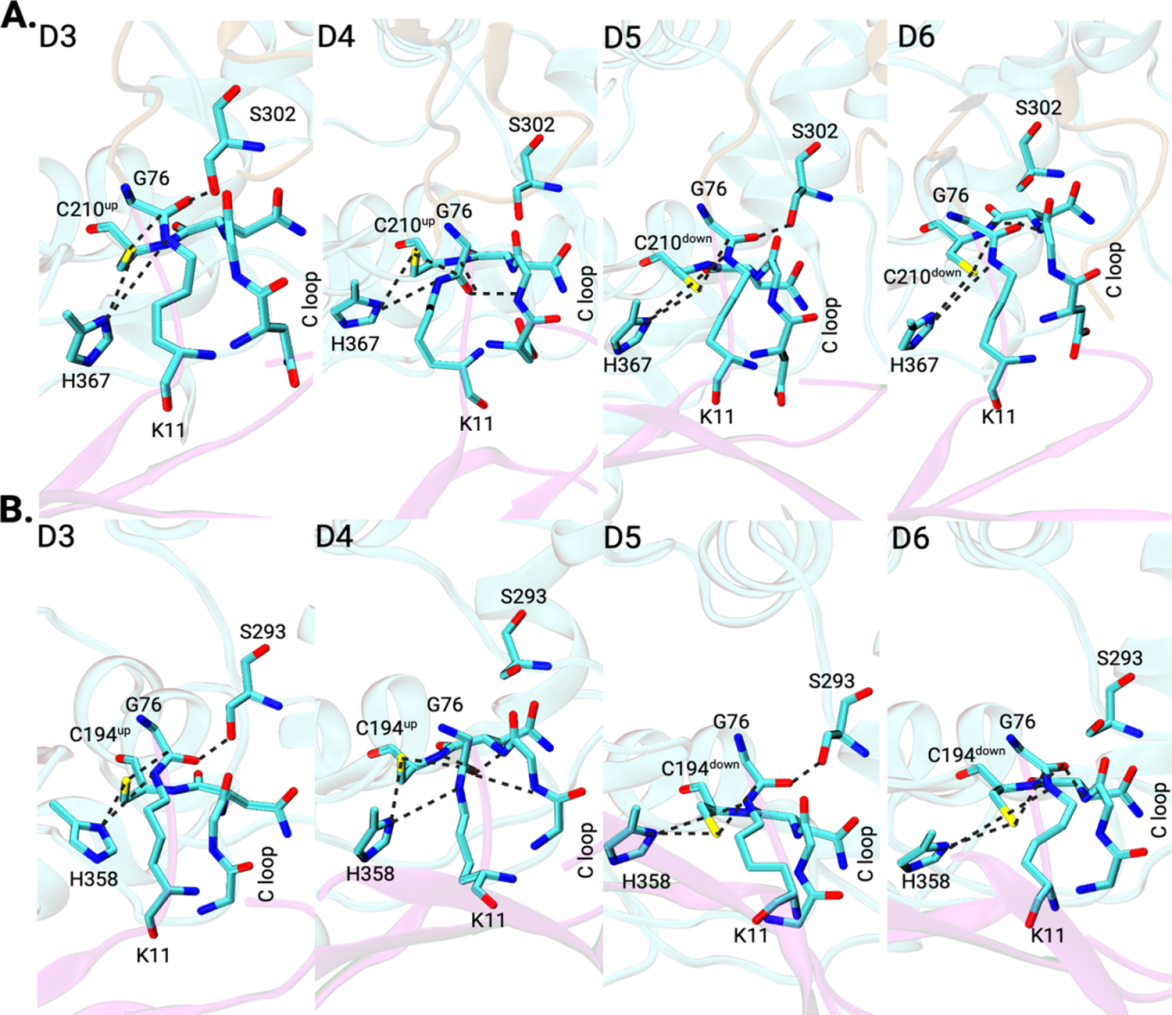
Representative structures of prereactive configurations “D” from MD simulations. All of them fulfill structural criteria in terms of distances and orientations for a nucleophilic attack and oxyanion stabilization (see text for details). Top: Cez2^0^Ub_2_ with different C210 side-chain conformations and hydrogen bond interactions with C-loop residues. Bottom: Cez^0^Ub_2_ with different C194 side-chain conformations and hydrogen bond interactions with C-loop residues.

Finally, configurations D3–D6 are a refinement of structures from configuration C and apply further filtering criteria to describe the structural arrangement of the C-loop and the formation of a hydrogen bond between the isopeptide carbonyl oxygen and the protein residue S302 (Figures 9 and 10). Figures S3 and S4 provide the sampling of productive configurations fulfilling the structural criteria of Table 3.

During the nucleophilic attack, the carbonyl carbon is displaced from its trigonal sp^2^ hybridization toward a more tetrahedral coordination. Finally, a covalent bond between the thiolate nucleophile and the electrophile carbon atom is formed, and a tetrahedral thioester intermediate is obtained. The negative charge is on the oxygen atom, which constitutes the “oxyanion” that needs to be stabilized by surrounding amino acid residues of the C-loop, i.e., the “oxyanion hole.” Cez2 residues G208 and C210(NH) are able to form hydrogen bonds with the substrate Ub(G76) carbonyl oxygen atom (Figure 9). The temporal evolution of these hydrogen bond interactions is given in Figure S8 for Cez2^0^Ub_2_ and Cez2^+/–^Ub_2_.

The oxyanion hole is perfectly prearranged in the D4 configurations of Cez2^0^Ub_2_ to stabilize the tetrahedral thioester intermediate. From configuration D4, only structures where the isopeptide carbonyl oxygen is oriented toward the C-loop and forms hydrogen bond interactions with the residues C210 and G208 were refined by QM/MM calculations. Hydrogen bond interactions between the diUb isopeptide carbonyl oxygen and the C-loop residues with interatomic distances of ≤0.4 nm were retained since they constitute the oxyanion hole and stabilize the tetrahedral intermediate. Figure S6 provides the histograms of the probability density distribution of sampling configuration D4. Long classical MD simulations are able to sample prereactive configurations for substrate-bound Cez2 and Cez, albeit with a low frequency (Table 4). In the case of neutral Cez2^0^Ub_2_, these configurations are observed for 0.01, 0.39, 0.48, and 0.49% of the simulation. The numbers for the zwitterionic charge state are very similar to 0.045, 0.13, 0.24, and 0.22%. The only structural differences between these configurations stem from (i) the side-chain conformations of C210 and (ii) the presence of a hydrogen bond between S302(Oγ) and G76(Oτ) atoms of the isopeptide bond (Figure 9). Since these differences are minor local structural alterations only, they can easily interconvert, and there is no need to consider all of them in QM/MM optimizations.

Cezanne-1 is the paralogue of Cezanne-2 and shows the same pronounced K11 selectivity. The structural dynamics of several intermediate states were investigated before by us.^32^ Here, we present new results from MD simulations of the Cez^0^Ub_2_ state in order to compare the sampling of prealigned catalytic site residues using the same distance and orientational criteria as for Cez2 (Figure S5). Configuration B was detected for 1.94% of the simulation time, and configuration C for 1.74%, indicating that a nucleophilic attack of C194(Sγ) to the G76-K11 isopeptide bond, followed by a proton transfer from H358(Nδ) to G76-K11 is feasible. Refinement of MD snapshots from configuration C yielded the presence of D3 (0.01%), D4 (0.93%), D5 (0.19%), and D6 (0.52%). They differed mainly in the side-chain conformation of C194 and the presence of hydrogen bonding between S293(Oγ) and G76(Oτ) of the isopeptide bond. In the D4 configuration of Cez, the E157(Cδ)···H358(Nε) distance fluctuated between 0.35 and 0.98 nm. The presence of water between E157 and H358 was detected in 76.88% of the D4 configuration structures. These findings suggest that also in Cezanne-1, there is a water-mediated H358–E157 indirect interaction that helps to stabilize the catalytic dyad.

Thus, our results show that Cezanne-1 and Cezanne-2 share the same reaction mechanism to perform selective proteolysis of K11-linked diUb.

### 3.6 QM/MM Refinement of diUb Cezanne-2 Enzyme–Substrate Complexes

Representative structures of the D4 cluster were taken from the MD simulations of Cez2^0^Ub_2_ (8 structures) and Cez2^+/–^Ub_2_ (6 structures) and subjected to QM/MM optimization (Figure 10). The E173(Cδ)···H367(Nε) distance varied between 0.45–0.92 nm in Cez2^0^Ub_2_ and 0.31–0.78 nm in Cez2^+/–^Ub_2_. Water molecules bridging E173 and H367 were detected in 96.75% of Cez2^0^Ub_2_ and 100% of the Cez2^+/–^Ub_2_ D4 configurations. This suggests the conservation of at least one water molecule in the aligned catalytic center of the potential productive configuration D4.

The optimized structures were analyzed regarding the structural parameters relevant for proteolytic catalysis, and the results support our MD findings and conclusions.

In seven of the eight optimized Cez2^0^Ub2 snapshots, the active site and substrate residues were aligned properly after QM/MM optimization and are in agreement with the cysteine protease mechanism (Table S1). All of these structures exhibited the identified E173–water–H367–C210 catalytic residue network, with Glu–water–His distances (angles) of 0.26–0.30 nm (149–176°) and His–Cys hydrogen bond distances of 0.33–0.36 nm (138–154°). The isopeptide bond carbonyl group of diUb and the G208(N) atom also formed a hydrogen bond with a distance (angle) of 0.28–0.30 nm (124–144°). In three structures, this carbonyl group also formed a hydrogen bond with the C210(N) backbone atom, which was at a distance (angle) of 0.36–0.39 nm (157–160°). The distance (angle) for the nucleophilic attack was 0.32–0.38 nm (80–105°). Two structures displayed a nucleophilic attack angle (80 and 85°), which is outside the defined range. However, as described below, these angles are not obstructing productivity. Thus, a proton transfer and subsequent nucleophilic attack leading to the tetrahedral intermediate stabilized by the C-loop residues are feasible also for these structures (Figure S4).

**Figure 11:**
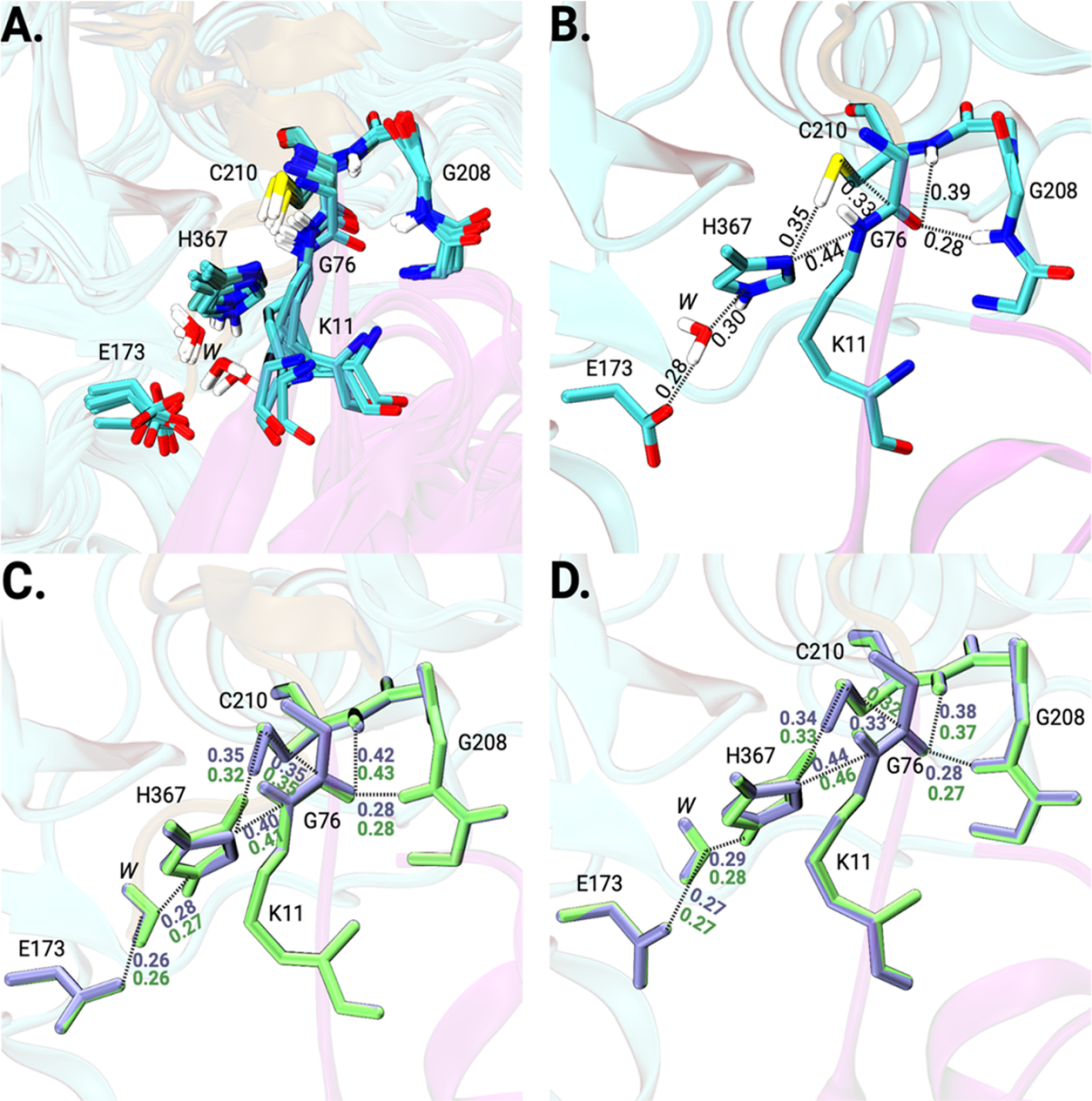
Examples of the QM/MM-optimized structures of Cezanne-2. (A) Seven superimposed Cez2^0^Ub2 structures identified as properly aligned and “productive.” (B) One example of a “prereactive” and productive Cez2^0^Ub_2_ snapshot with relevant interatomic distances in nm (structure 7 in Table S1). (C, D) Two examples of the QM/MM-refined structure of Cez2^0^Ub_2_ (ice blue) and Cez2^+/–^Ub_2_ (lime) structures for which proton transfer energies were calculated. Relevant interatomic distances are provided (in nm).

For further assessment, we calculated the reaction energy of the proton transfer reaction for two selected Cez2^0^Ub2 structures with aligned active sites. We manually moved the proton from C210 to H367 and reoptimized the structures (Figure 11). This resulted in Cez2^+/–^Ub_2_ structures with relative energies of −4.6 and −0.2 kcal/mol compared to Cez2^0^Ub2, which indicated that proton transfer is thermodynamically feasible (Table S2). The incorporation of dispersion corrections and use of a larger basis set gave proton transfer energies of −3.8 and 0.8 kcal/mol and confirmed the slight exothermicity or close to thermoneutrality of this event. M06-2X single point calculations were also in agreement and gave a consistent picture (Table S2). Upon proton transfer, the nucleophilic attack angle of 80° in Cez^0^Ub_2_ shifted to 88° in the Cez2^+/–^Ub_2_ structure and thus is closer to the ideal value of 107°. A similar angle shift, from 92 to 98°, was observed for the second structure (Table S2). This shows that the QM/MM refined structure of Cez2^+/–^Ub_2_ is properly aligned for the following nucleophilic attack to the carbonyl carbon of the isopeptide bond.

Such a shift in the Bürgi–Dunitz angle could also be sampled during the MD simulations (Figure S4). These structural changes, plus the calculated reaction energies, made us consider the Cez2^0^Ub2 structures with nucleophilic attack angles of 80 and 85° to be “productive.” These results show the importance of using QM/MM calculations to refine carefully chosen MD structures in investigating enzyme reactions. Thus, structural and energetic QM/MM data support the proposed reaction mechanism of Cez2 catalysis.

On the other hand, one of the optimized Cez2^0^Ub_2_ structures was not “productive” and gave structural changes in the active site (Table S1; structure 8). Here, C210–H367 proton transfer was not feasible, as C210(Sγ) and H367(Nδ) during the optimization converged back to an interatomic distance (angle) of 0.41 nm (109°). After QM/MM refinement, the nucleophilic attack angle was 66°, which is far from the ideal BD angle of 107°. In contrast, H367 and E173 formed a strong direct hydrogen bond at a distance (angle) of 0.29 nm (170°) and did not show the water-mediated indirect interaction. This is another example of the importance of QM/MM refinement of MD structures, as in the MD structure, the nucleophilic attack angle was 107°, and both inter-residue interactions were formed as expected. Both MD simulations and QM/MM calculations agree that the direct E–H–C catalytic triad gives a catalytically incompetent configuration.

Similar conclusions can be drawn for the QM/MM-optimized Cez2^+/–^Ub_2_ MD snapshots (Figure 11). Four of the six optimized structures were “productive,” according to the definition above (Table S3). In these structures, the isopeptide bond carbonyl carbon formed either one or two hydrogen bonds with the C-loop. C210^–^ and H367^+^ formed a hydrogen bond with a distance (angle) of 0.32–0.34 nm (160–173°). The nucleophilic attack distance (angle) was 0.34 nm (92–115°). The E-water–H–C catalytic network was present in three of the structures, with either 1 or 2 waters bridging H367 and E173, while it was absent in one structure. In the latter, the histidine residue was rotated, and the H367(Nε) atom formed a hydrogen bond with the carbonyl oxygen of Thr204(O) instead, with a distance (angle) of 0.27 nm (148°). However, as discussed in the previous sections, apart from typical protease reaction criteria, the most critical structural requirement for a “productive” configuration is the presence of the E-*W*-H water bridge and its role in H367 polarization.

## 4 Conclusions

The OTU deubiquitinylase Cezanne-2 is a cysteine protease with a pronounced selectivity for K11-linked polyubiquitin chains. Using a combined approach of protein structural modeling, extensive sampling of several charge states and reaction intermediates by all-atom MD simulations, and QM/MM refinement of MD snapshots, structural details of the enzyme activation and reaction were elucidated. In the absence of a substrate, there is an equilibrium between active site residues cysteine and histidine in their neutral and zwitterionic charge states. Substrate binding correctly positions the cysteine and histidine residues, enhances the short inter-residue distances between these residues, shifts the equilibrium toward the charge-separated state, and thus activates the OTU enzyme. The third catalytic site residue, glutamate, does not make direct interactions with the nearby histidine. Rather, its interaction is mediated by a strictly conserved water molecule that is present for >90% of the simulation in all Cezanne-2 states.

This shows that Cezanne-2 uses a catalytic dyad of cysteine/histidine residues to cleave the isopeptide bond of K11-linked diubiquitin. The role of the third residue is as follows: on the one hand, it stabilizes the orientation of the dyad residues, and the bridging water molecule might affect the p*K*_b_ values of histidine. Glutamate also establishes persistent electrostatic interactions with the Lys33 residue of the substrate and, thus, assists substrate recognition and binding.

Proteolytic cleavage of the diubiquitin isopeptide bond requires a close approach and correct orientation of the enzyme catalytic residues with respect to the substrate carbonyl group. Apart from thresholds for inter-residue distances, an approach of the thiolate nucleophile in an orientation close to an optimal Bürgi–Dunitz angle is necessary. Further structural definitions of prereactive configurations involve dihedral angle orientations of the cysteine thiol and stabilization of the tetrahedral oxyanion intermediate by residues of the C-loop. Since classical force fields are parametrized to describe equilibrium structures of (bio)molecular systems, these nonstandard binding situations are rarely sampled. Orbital orientation, polarization effects, and changes from trigonal planar to tetrahedral hybridization are not well represented. However, quantum chemical refinement of selected MD snapshots by QM/MM calculations of the enzyme–substrate complex provides reliable structural parameters that are in full agreement with the proteolytic mechanism.

Structural resolution of enzyme–substrate complexes is challenging since enzymatic turnover must be inhibited to co-crystallize the substrate in or close to the catalytic site. For cysteine proteases, activity-based probes or mutations in the active site are sometimes used to characterize a complex that is close but not identical with a physiological binding situation. In addition, mutations of the catalytic cysteine residue to a nonreactive alanine, for example, do not provide the full-resolution enzyme–substrate picture. However, these artifacts must be recovered, and a refinement by MD simulations is required. Likewise, protein–protein complex structural models from deep learning approaches have to be carefully inspected, and further MD refinement steps may also be necessary. The detection and interpretation of subtle differences in inter-residue distances (in order to issue statements about the state of enzyme activation) or substrate binding and orientation (to ensure a physiological binding situation) require curation by an expert scientist in this field.

## Data Availability

All files to reproduce the simulations: PDB, PSF, topology, and OpenMM and scripts are freely available from zenodo at https://doi.org/10.5281/zenodo.13972885.

## Supporting Information Available

Additional details such as RMSD plots, structural details of selected MD frames, and criteria for selection; MD sampling of reactive configurations. (PDF)
