# Supplementary material for: Activation and Reactivity of the Deubiquitinylase OTU Cezanne-2 from MD Simulations and QM/MM Calculations
Source: J Chem Inf Model. 2025 Jan 9;65(2):921–36. doi: 10.1021/acs.jcim.4c01964 (PMC11776055; doi:10.1021/acs.jcim.4c01964)
Supplement: Supplementary file 1 — ci4c01964_si_001.pdf [file ci4c01964_si_001.pdf]

Supplementary Information

Activation and reactivity of the deubiquitinylase OTU Cezanne-2 from MD simulations and QM/MM calculations

Metehan Ilter[1], Andrés M. Escorcia[1], Eric Schulze-Niemand[1,2], Michael Naumann[2], Matthias Stein[1*]

[1] Max Planck Institute for Dynamics of Complex Technical Systems, Molecular Simulations and Design Group, Sandtorstrasse 1, 39106 Magdeburg, Germany.

[2] Institute for Experimental Internal Medicine, Medical Faculty, Otto von Guericke University, Leipziger Straße 44, 39120 Magdeburg, Germany

*Corresponding author: matthias.stein@mpi-magdeburg.mpg.de

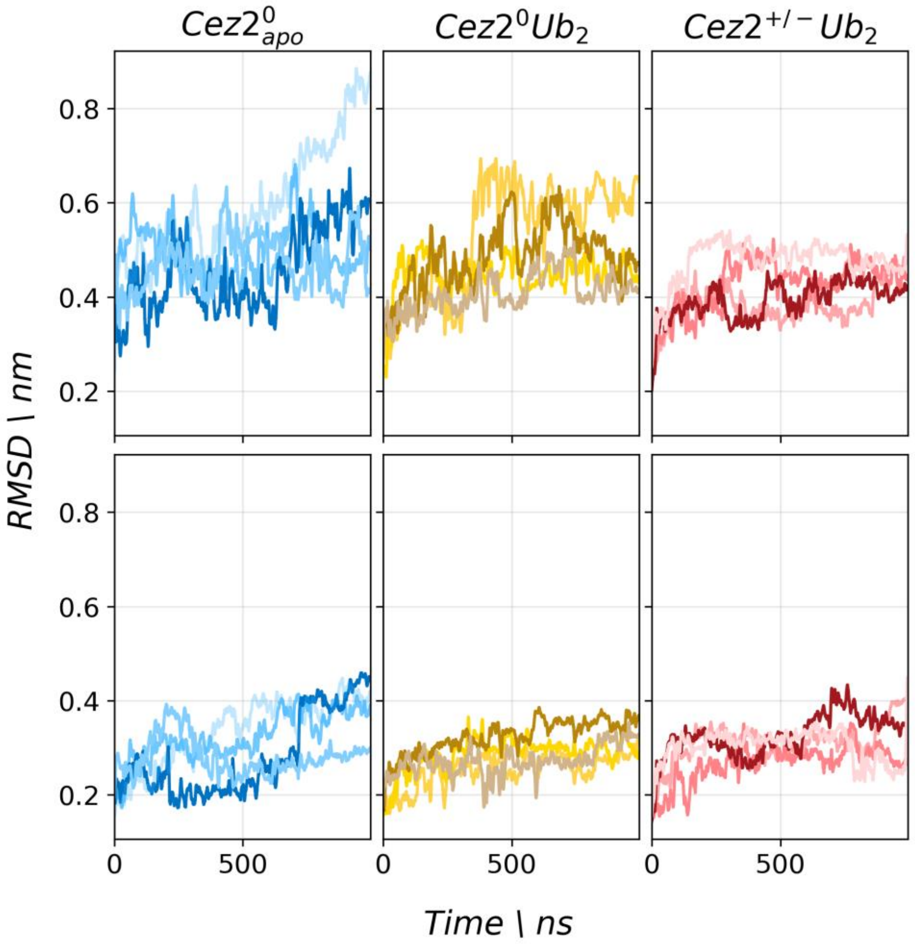

Figure S1. Cα RMSD of the OTU domains of $Cez2^0_{apo}$, $Cez2^0Ub_2$, and $Cez2^{+/-}Ub_2$ from **(top)** entire initial structures and **(bottom)** when disordered loops are excluded with respect to their initial structural. Four individual trajectories of 1 µs of each system are shown as moving averages with a window size of 50.

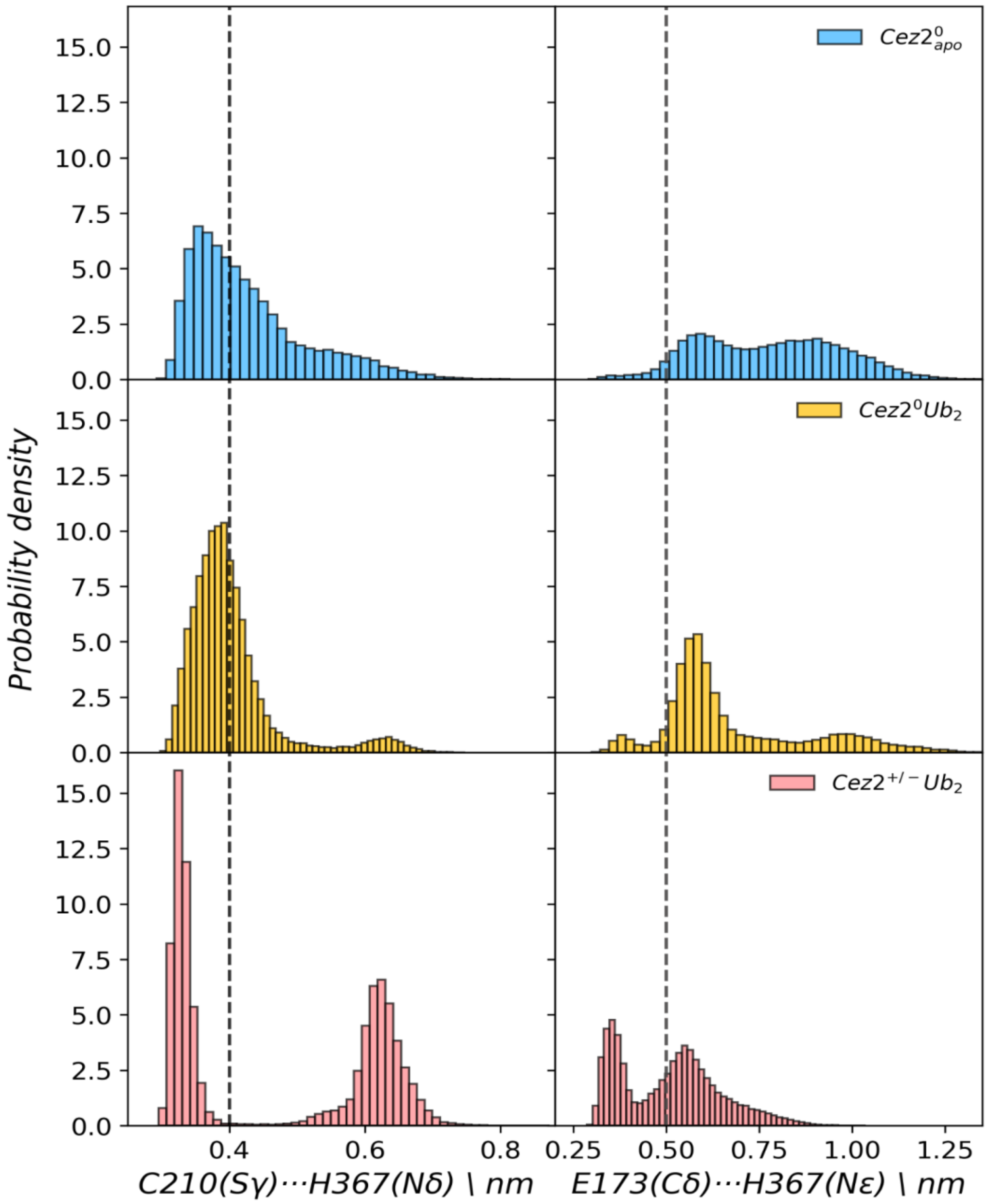

Figure S2. Alignment of the catalytic center residues upon substrate binding – The histograms on the left show the probability densities of C210(Sγ)···H367(Nδ) distances over the pooled trajectories of $Cez2^0$apo (blue), $Cez2^0Ub_2$ (yellow), and $Cez2^{+/-}Ub_2$ (pink) trajectories. The histograms on the right illustrate the probability densities of E173(Cδ)···H367(Nε) distances of the studied systems. The dashed lines at 0.4 and 0.5 nm correspond to the cut-off distances for the direct proton transfer between C210-H367 and E173-H367, respectively.

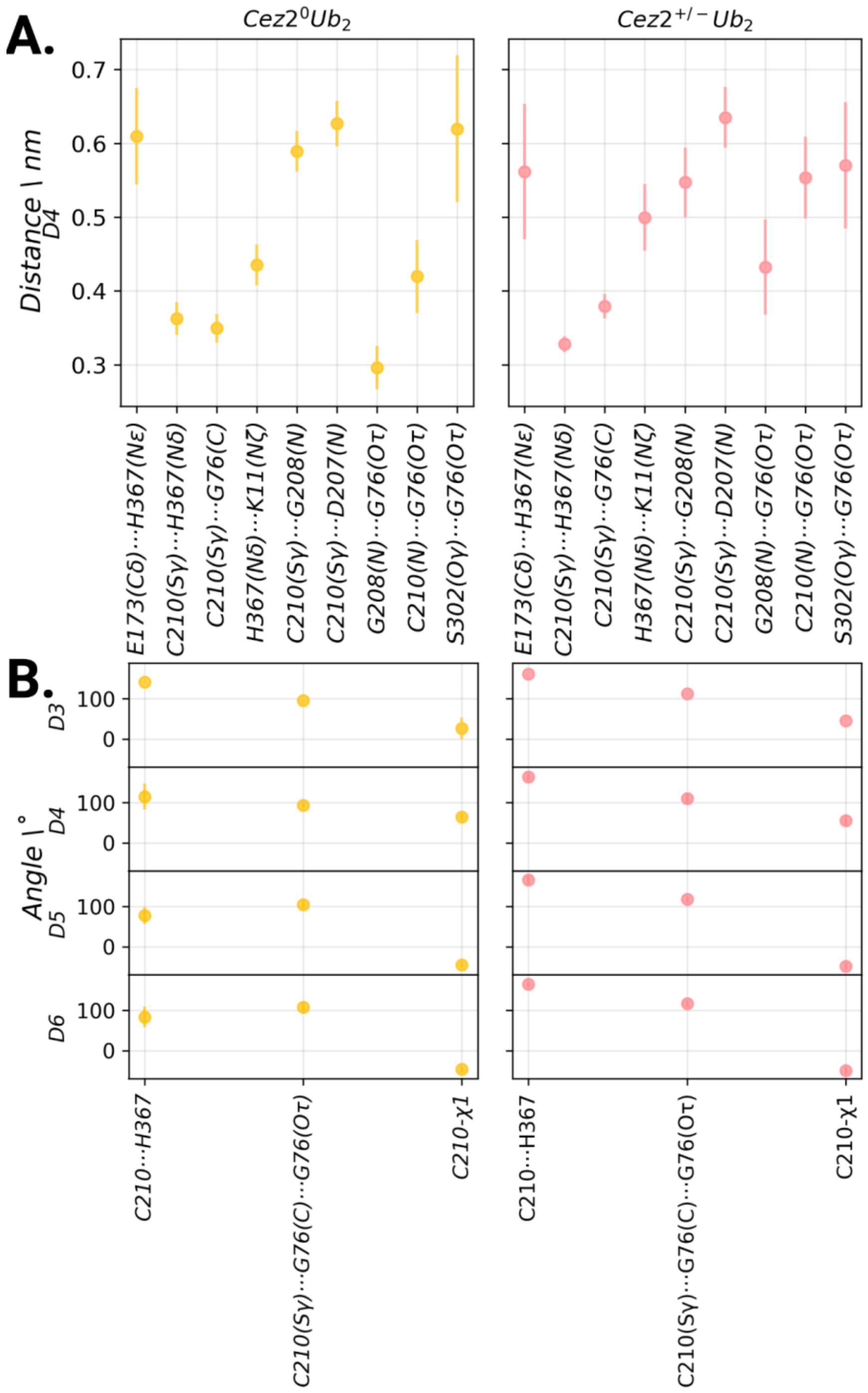

Figure S3. MD sampling of productive configurations. The dot plots show the average and standard deviations of **A.** relevant interatomic distances and **B.** angles for the catalytic process involving direct proton transfer between substrate and the catalytic C210 and H367 residues. Yellow and pink dots, along with error bars, represent data obtained from MD snapshots of $Cez2^0Ub_2$ and $Cez2^{+/-}Ub_2$, respectively. These snapshots were selected based on their adherence to the geometrical descriptors of configurations defined in **Table 2**. D3, D4, D5, and D6 configurations were detected in 0.005%, 0.39%, 0.47%, and 0.49% of the simulation time of $Cez2^0Ub_2$, whereas these configurations are sampled in 0.05%, 0.13%, 0.24%, and 0.22% of the total simulation time of $Cez2^{+/-}Ub_2$.

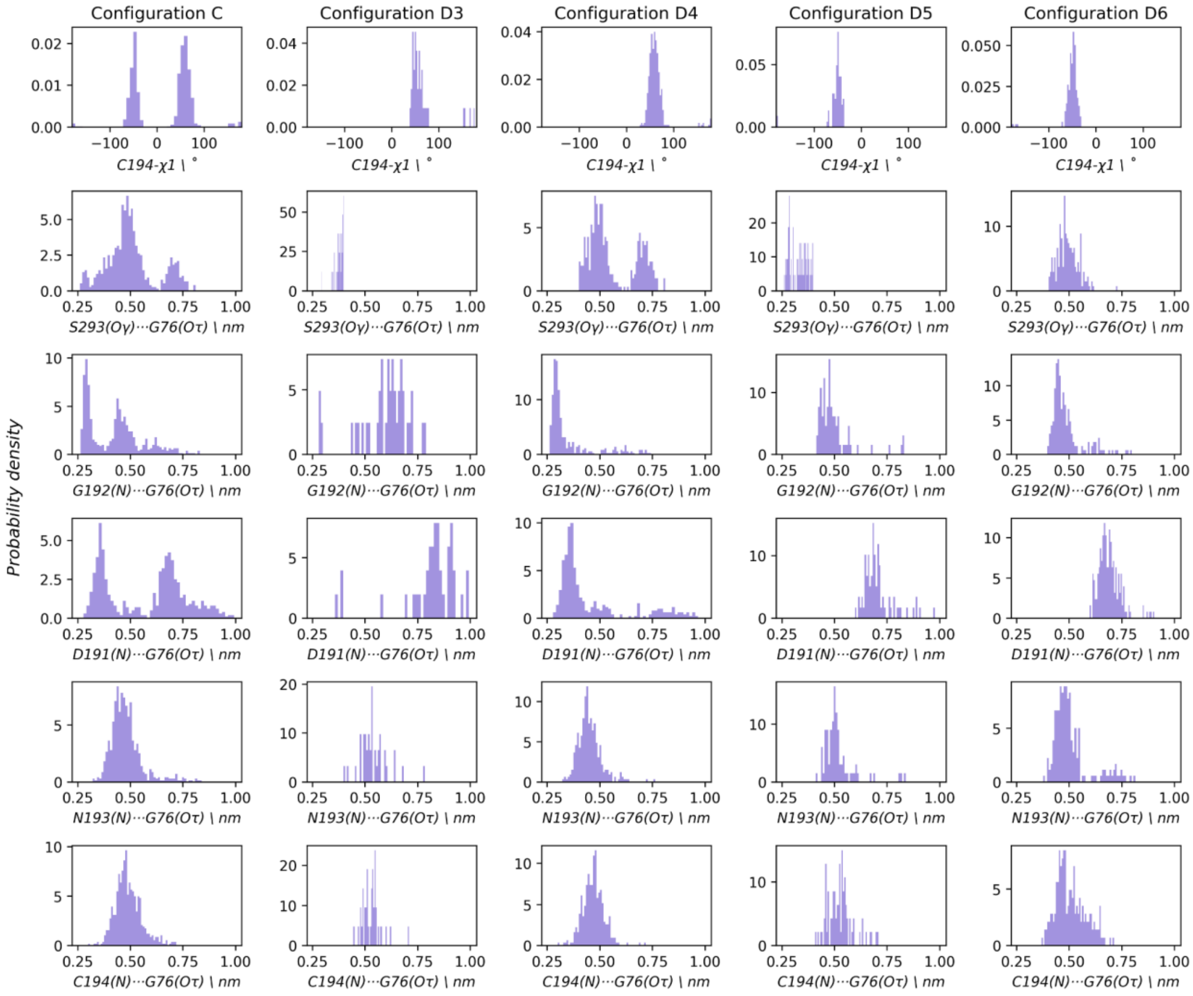

Figure S4. MD sampling of C-loop residues in productive configurations (C, D3, D4, D5, and D6) of Cez0Ub2. Histograms display the distribution of key geometrical descriptors: C194-χ1 dihedral angle, S293(OG)···G76(OT), G192(N)···G76(Oт), D191(N)···G76(Oт), N193(N)···G76(Oт), and C194(N)···G76(Oт) the. Probability densities are calculated by dividing date into 50 bins.

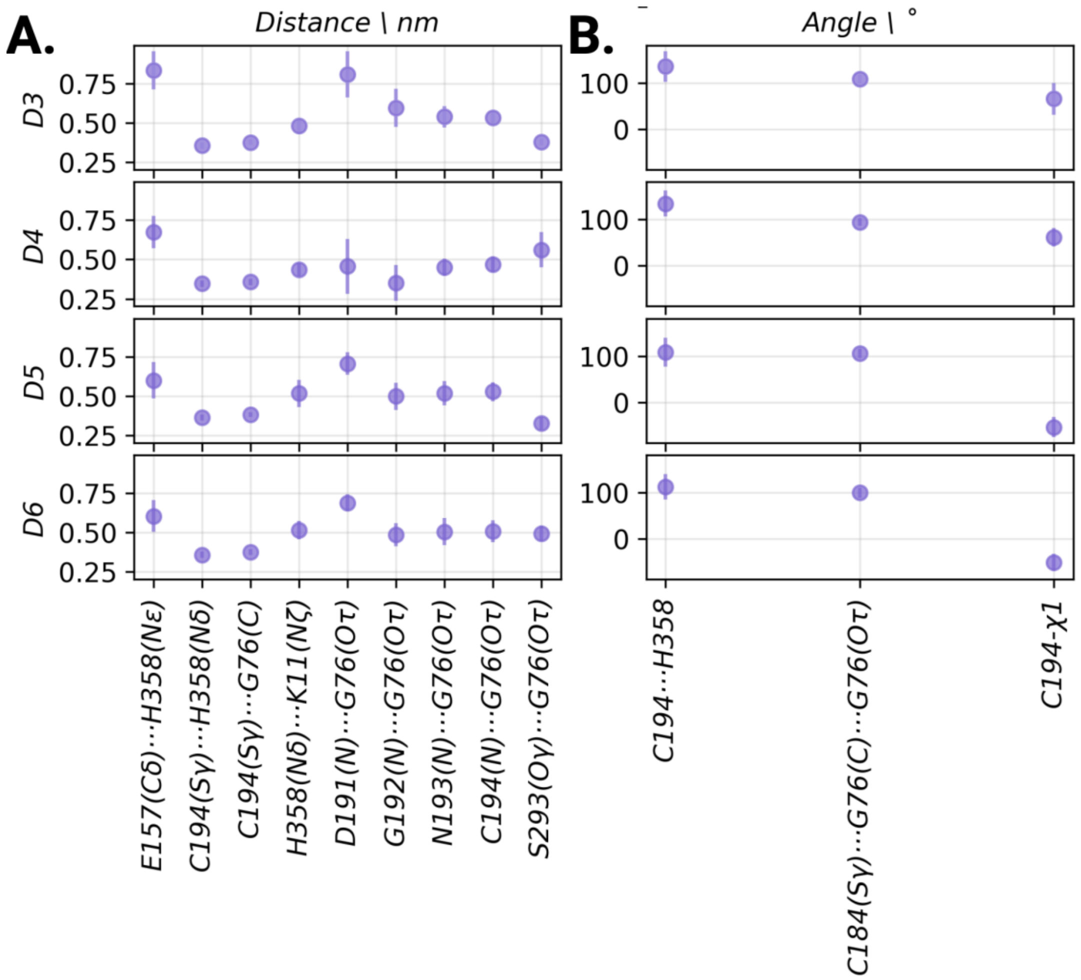

Figure S5. Productive configurations of Cez⁰Ub₂. The dot plots show the average and standard deviations of **A.** relevant interatomic distances and **B.** angles for the catalytic process involving direct proton transfer between substrate and the catalytic C194 and H358 residues. These snapshots were selected based on their adherence to the geometrical descriptors of configurations defined in **Table 2**. D3, D4, D5, and D6 configurations were detected in 0.01%, 0.93%, 0.19%, and 0.52% of the simulation time of Cez20Ub2.

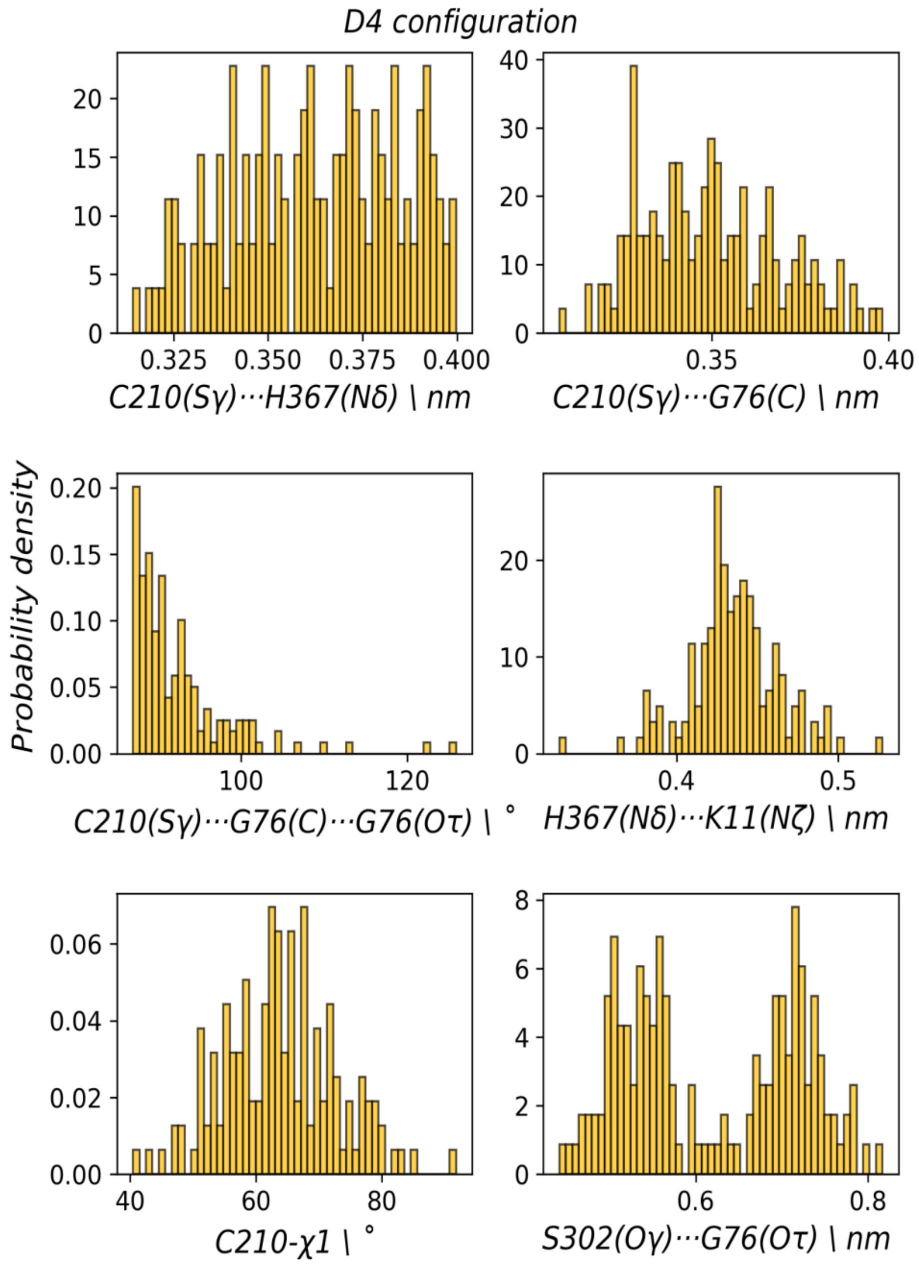

Figure S6. Geometrical descriptors for D4 configuration for Cez2^{0}Ub$_{2}$ – Histograms show the probability density distributions of the geometrical descriptors used to identify D4 configuration, including C210(Sγ)···H367(Nδ), C210(Sγ)···G76(C), H367(Nδ)···K11(Nζ), and S302(Oγ)···G76(Oτ) distances as well as C210(Sγ)···G76(C)···G76(Oτ) and C210-χ1 angles in Cez20Ub2 trajectory. The probability densities are estimated by dividing timeline data into 50 equidistant bins.

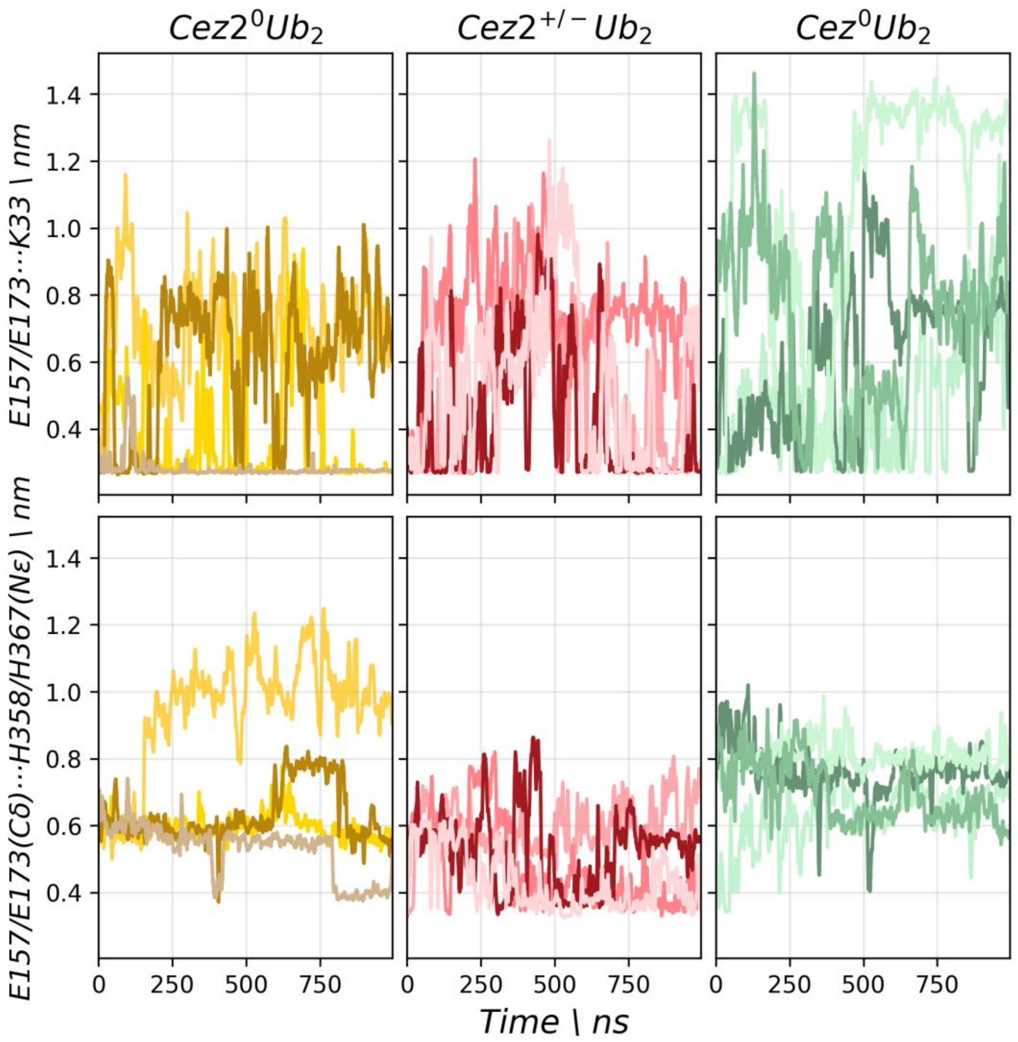

Figure S7. Temporal evolution of the inter-residue distances of the active site residues E-H and E-K. The line plots show the moving average of measured distances with a window range of 50.

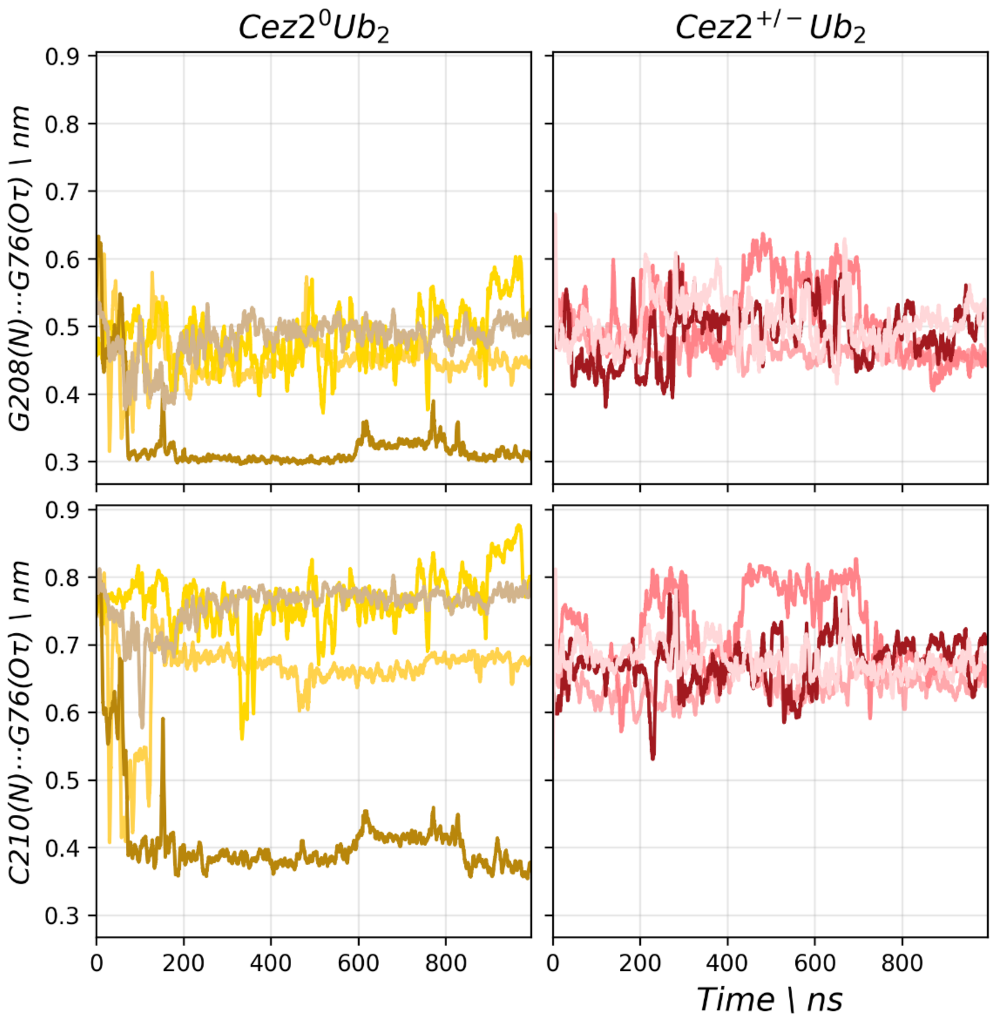

Figure S8. Temporal evolution of inter-residue distances of Cez2 to stabilize the hydroxide intermediate of ubiquitin in the neutral and zwitterionic charge states (the oxyanion hole). Top: monitoring of Cez2(G208(N)) and Ub(G76(Oτ)) distance. Bottom: monitoring of the C210(N) and G76(Oτ). The line plots show the moving average of measured distances with a window range of 50.

Table S1. Relevant interatomic distances (nm) and angles (°) observed in the QM/MM optimized D4 structures of Cez2^{0}Ub$_{2}$[a]

Structure[b]	E-Water-H-C hydrogen bond network		Nucleophilic attack and subsequent proton transfer to K11		C-loop configuration			Hydrogen bond interactions with the oxyanion hole	
	C210(Sγ)-H367(Nδ)	E173(Oε)-*W(O)*-H367(Nε)[c]	C210(Sγ)-G76(C)	H367(Nδ)-K11(Nζ)	C210-χ1	C210(Sγ)-D207(N)	C210(Sγ)-G208(N)	C210(N)-G76(Oτ)	G208(N)-G76(Oτ)
1	0.34 (153)	0.27 (174), 0.28 (163)	0.38 (105)	0.42	51	0.60	0.56	0.52 (163)	0.30 (139)
2	0.34 (144)	0.27 (167), 0.28 (155)	0.34 (95)	0.40	58	0.60	0.57	0.44 (157)	0.28 (144)
3	0.33 (154)	0.27 (172), 0.29 (165)	0.34 (92)	0.42	57	0.59	0.56	0.44 (166)	0.28 (124)
4	0.35 (138)	0.26 (176), 0.28 (151)	0.35 (92)	0.40	59	0.61	0.58	0.42 (158)	0.28 (142)
5	0.34 (146)	0.27 (167), 0.29 (149)	0.33 (80)	0.44	60	0.59	0.56	0.38 (158)	0.28 (141)
6	0.36 (145)	0.27 (172), 0.28 (159)	0.32 (94)	0.43	62	0.65	0.61	0.36 (157)	0.29 (136)
7	0.35 (144)	0.28 (168), 0.30 (159)	0.33 (85)	0.44	57	0.60	0.58	0.39 (160)	0.28 (135)
8	0.41 (109)	*0.29 (170)*[d]	0.36 (66)	0.51	60	0.62	0.58	0.36 (154)	0.29 (148)

[a] For convenience, the angles for hydrogen bonds and the nucleophilic attack are given in parentheses next to the interatomic distances.

[b] There are three major groups of optimized structures: i) productive structures where the carbonyl oxygen of the diUb (iso)peptide bond is forming one (1) hydrogen bond with the C-loop (purple background), ii) productive structures where the (iso)peptide bond carbonyl oxygen forms two (2) hydrogen bonds with the C-loop (magenta background), and iii) a non-productive structure where E173 and H367 are forming a hydrogen bond interaction (i.e. no bridging water, grey background). See the main text for more details.

[c] *W:O* corresponds to the oxygen atom of the water molecule bridging E173 and H367 in structures 1-7. Data on the left corresponds to the E173-*Water* interaction while the data on the right to the H367-*Water* interaction.

[d] Value corresponds to a hydrogen bond interaction (i.e. no bridging water) between E173 and H367.

Table S2. Relevant interatomic distances (nm) and angles (°) of the QM/MM optimized Cez2^{0}Ub2 and Cez2$^{+/-}$Ub2 structures from which proton transfer energies C210···H367 were calculated[a]

Reaction System	Chemical Species	E-Water-H-C hydrogen bond network		Nucleophilic attack and subsequent proton transfer to K11		Hydrogen bond interactions with the oxyanion hole		ΔE B3LYP/B3LYP-D3/M06-2X
		**C210(Sγ)-H367(Nδ)**	**E173(Oε)-*W(O)*-H367(Nε)[b]**	**C210(Sγ)-G76(C)**	**H367(Nδ)-K11(Nζ)**	**C210(N)-G76(Oτ)**	**G208(N)-G76(Oτ)**	
1	Cez2^{0}Ub2	0.35 (138)	0.26 (176), 0.28 (151)	0.35 (92)	0.40	0.42 (158)	0.28 (142)	-0.2/0.8/-0.4
	Cez2^{+-}Ub2	0.32 (157)	0.26 (175), 0.27 (155)	0.35 (98)	0.41	0.43 (150)	0.28 (145)	
2	Cez2^{0}Ub2	0.34 (146)	0.27 (167), 0.29 (149)	0.33 (80)	0.44	0.38 (158)	0.28 (141)	-4.6/-3.8/-5.7
	Cez2^{+-}Ub2	0.33 (162)	0.27 (167), 0.28 (151)	0.32 (88)	0.46	0.37 (156)	0.27 (145)	

[a] For convenience, angles (for hydrogen bonds and the nucleophilic attack) are given in parentheses.

[b] *W(O)* corresponds to the oxygen atom of the water molecule bridging E173 and H367. Data on the left correspond to the E173-*Water* interaction while the data on the right to the H367-*Water* interaction.

Table S3. Relevant interatomic distances (nm) and angles (°) observed in the QM/MM optimized D4 structures of Cez2^{+}-Ub$_2$[a]

Structure[b]	E-Water-H-C hydrogen bond network		Nucleophilic attack and subsequent proton transfer to K11		C-loop configuration			Hydrogen bond interactions with the oxyanion hole	
	C210(Sγ)-H367(Nδ)	E173(Oε)-*W(O)*$_{(1-2)}$-H367(Nε)[c]	C210(Sγ)-G76(C)	H367(Nδ)-K11(Nζ)	C210-χ1	C210(Sγ)-D207(N)	C210(Sγ)-G208(N)	C210(N)-G76(Oτ)	G208(N)-G76(Oτ)
1	0.32 (162)	0.28 (174), 0.28 (154)	0.40 (115)	0.50	48	0.53	0.50	0.61 (169)	0.37 (102)
2	0.32 (170)	0.28 (170), *0.28 (157)*, 0.28 (147)	0.34 (92)	0.42	52	0.67	0.59	0.46 (168)	0.31 (106)
3	0.32 (160)	*0.45*[d]	0.40 (102)	0.45	68	0.64	0.64	0.45 (150)	0.29 (151)
4	0.34 (173)	0.27 (171), 0.29 (172)	0.34 (95)	0.45	69	0.66	0.63	0.36 (154)	0.29 (130)
5	0.32 (158)	0.28 (170), 0.27 (152)	0.43 (119)	0.50	46	0.60	0.54	0.63 (178)	0.38 (70)
6	0.32 (167)	0.28 (165), *0.29 (169)*, 0.28 (171)	0.41 (103)	0.44	51	0.66	0.56	0.59 (168)	0.38 (72)

[a] For convenience, the angles for hydrogen bonds and the nucleophilic attack are given in parentheses next to the interatomic distances.

[b] There are three major groups of optimized structures: i) productive structures where the carbonyl oxygen of the diUb (iso)peptide bond is forming one (1) hydrogen bond with the C-loop (purple background), ii) a productive structure where the (iso)peptide bond carbonyl oxygen forms two (2) hydrogen bonds with the C-loop (magenta background), and iii) non-productive structures (distances for nucleophilic attack of > 0.40 nm & no formation of hydrogen bonds with the oxyanion hole, grey background).

[c] *W:O* corresponds to the oxygen atom(s) of the water(s) molecule(s) bridging E173 and H367 (1-2: one or two water molecules). The data on the left corresponds to the E173-*Water* interaction, *the data on the centre (for when there are two bridging waters) to the Water-Water interaction*, and the data on the right to the H367-*Water* interaction.

[d] Interatomic distance between E173:Oε and H367:Nε. Neither a hydrogen bond interaction nor a water bridge is formed between these residues. Instead, a strong hydrogen bond interaction between H367:Nε and the carbonyl oxygen of Thr204 is observed, with a distance (angle) of 0.27 nm (148°).
